# Supplementary material for: Phosphorylated lipid-conjugated oligonucleotide selectively anchors on cell membranes with high alkaline phosphatase expression
Source: Nat Commun. 2019 Jun 20;10:2704. doi: 10.1038/s41467-019-10639-6 (PMC6586821; doi:10.1038/s41467-019-10639-6)
Supplement: Supplementary file 1 — Supplementary Information [file 41467_2019_10639_MOESM1_ESM.pdf]

Supplementary Information

Phosphorylated Lipid-Conjugated Oligonucleotide Selectively Anchors on Cell Membrane with High Alkaline Phosphatase Expression

Jin et al.

Supplementary Methods

Materials. For organic synthesis, all reagents were purchased commercially without further treatment. For DNA synthesis, base and labeled reagents were purchased from Glen Research. Bis(2-cyanoethyl)-N,N-diisopropyl phosphoramidite, a chemical phosphorylation reagent, was purchased from Sigma-Aldrich. Alkaline phosphatase, cell lysis buffer for Western and IP without inhibitors, BCA Protein Assay Kit, Alkaline Phosphatase Assay Kit and the cell membrane probe (DiO) were purchased from Beyotime Biotechnology. Ultrapure deionized water was used in all experiments, excluding organic synthesis, and was obtained from a Milli-Q Biocel system.

Synthesis of C6 phosphoramidite. The synthesis procedure of C6 phosphoramidite refers to the previous report.[1] Briefly, 1-bromohexane (2.0 g, 12.1 mmol), N,N-diisopropylethylamine (DIPEA) (3.10 g, 24.0 mmol) and *p*-aminophenol (0.66 g, 6.0 mmol) were dissolved in 15 mL anhydrous N,N-dimethylformamide (DMF). The reaction was allowed to reflux under the protection of nitrogen gas and monitored by thin-layer chromatography (TLC). When the reaction was completed, the mixture was diluted with 100 mL dichloromethane and washed successively with saturated $NaHCO_3$ and brine. The organic layer was collected and dried by anhydrous Na_2SO_4. The solvent was removed by rotary evaporator. After purification through a flash chromatography column, 4-(dihexylamino)phenol was obtained as a colorless solid.

One gram 4-(dihexylamino)phenol (3.6 mmol) and DIPEA (0.93 g, 7.2 mmol) were dissolved in 30 mL anhydrous dichloromethane. The reaction was allowed to cool on ice bath under the protection of nitrogen gas. Then, 2-cyanoethyl N,N-diisopropyl-chlorophosphoramidite (0.85 g, 3.6 mmol) was added dropwise. The ice bath was removed, and the reaction was stirred for an additional one hour. When the reaction was completed, 100 mL dichloromethane were added, and the mixture was washed successively by saturated $NaHCO_3$, brine and water. The organic layer was collected and dried by anhydrous Na_2SO_4. The solvent was removed by rotary evaporator. After purification through a flash chromatography column, 1.0 g C6 phosphoramidite was obtained as a colorless oily liquid. ^{1}H NMR (400 MHz, $CDCl_3$): δ 6.89 (d, J=8.3 Hz,

2H), 6.56 (d, J=8.2 Hz, 2H), 3.99-3.83 (m, 2H), 3.72 (tt, J=13.4, 6.8 Hz, 2H), 3.17 (t, J=7.4 Hz, 4H), 2.65 (t, J=6.4 Hz, 2H), 1.53 (s, 4H), 1.30 (s, 12H), 1.21 (dd, J=14.5, 6.7 Hz, 12H), 0.89 (d, J=6.1 Hz, 6H). ^{31}P NMR (162 MHz, $CDCl_3$): δ 146.9. ^{13}C NMR (101 MHz, $CDCl_3$): δ 144.59, 144.26, 120.94, 117.60, 113.28, 58.94, 58.76, 51.62, 43.66, 43.53, 31.77, 27.24, 26.91, 24.72, 24.65, 24.46, 24.39, 22.71, 20.33, 14.07. The NMR spectra were shown in Supplementary Figure 28-30.

Synthesis of C9 phosphoramidite. The synthesis procedure of C9 phosphoramidite is same as that of C6 phosphoramidite. ^{1}H NMR (400 MHz, $CDCl_3$): δ 6.89 (d, J=8.3 Hz, 2H), 6.55 (d, J=8.2 Hz, 2H), 4.03-3.83 (m, 2H), 3.72 (m, 2H), 3.17 (t, J=7.4 Hz, 4H), 2.65 (t, J=6.4 Hz, 2H), 1.52 (s, 4H), 1.27 (s, 24H), 1.21 (dd, J=14.6, 6.7 Hz, 12H), 0.88 (t, J=6.2 Hz, 6H). ^{31}P NMR (162 MHz, $CDCl_3$): δ 146.93. ^{13}C NMR (101 MHz, $CDCl_3$): δ 144.60, 144.26, 120.93, 120.85, 117.60, 113.27, 58.94, 58.76, 51.62, 43.66, 43.53, 31.90, 29.65, 29.57, 29.30, 27.27, 27.25, 24.72, 24.65, 24.46, 24.39, 22.68, 20.33, 14.12. The NMR spectra were shown in Supplementary Figure 31-33.

Synthesis of C12 phosphoramidite. The synthesis procedure of C12 phosphoramidite is same as that of C6 phosphoramidite. ^{1}H NMR (400 MHz, $CDCl_3$): δ 6.89 (d, J=8.4 Hz, 2H), 6.55 (d, J=8.3 Hz, 2H), 3.99-3.82 (m, 2H), 3.72 (tt, J=13.4, 6.8 Hz, 2H), 3.17 (t, J=7.4 Hz, 4H), 2.65 (t, J=6.5 Hz, 2H), 1.52 (s, 4H), 1.26 (s, 36H), 1.21 (dd, J=14.7, 6.8 Hz, 12H), 0.88 (t, J=6.3 Hz, 6H). ^{31}P NMR (162 MHz, $CDCl_3$): δ 146.9. ^{13}C NMR (101 MHz, $CDCl_3$): δ 144.59, 144.26, 120.93, 120.85, 117.59, 113.27, 58.94, 58.76, 51.62, 43.66, 43.53, 31.94, 29.70, 29.69, 29.66, 29.58, 29.37, 27.28, 27.26, 24.71, 24.64, 24.46, 24.39, 22.71, 20.39, 20.33, 14.13. The NMR spectra were shown in Supplementary Figure 34-36.

Synthesis of C15 phosphoramidite. The synthesis procedure of C15 phosphoramidite is same as that of C6 phosphoramidite. ^{1}H NMR (400 MHz, $CDCl_3$): δ 6.89 (d, J=8.4 Hz, 2H), 6.55 (d, J=8.4 Hz, 2H), 4.01-3.84 (m, 2H), 3.72 (tt, J=13.4, 6.7 Hz, 2H), 3.26-3.10 (m, 4H), 2.66 (t, J=6.5 Hz, 2H), 1.52 (s, 4H), 1.26 (s, 48H), 1.21 (dd, J=14.9, 6.8 Hz, 12H), 0.88 (t, J=6.4 Hz, 6H). ^{31}P NMR (162 MHz, $CDCl_3$): δ 146.9. ^{13}C NMR (101 MHz, $CDCl_3$): δ 144.59, 144.17, 120.92, 120.84, 117.58, 113.26, 58.94, 58.75, 51.62, 43.66, 43.53, 31.94, 29.70, 29.68, 29.64, 29.58, 29.37, 27.28, 27.26, 24.71, 24.64, 24.46,

24.39, 22.70, 20.39, 20.32, 14.13. The NMR spectra were shown in Supplementary Figure 37-39.

FL measurements of Nile Red-encapsulated oligonucleotides. The corresponding oligonucleotides were diluted with TBS buffer to the final concentration of 10 μM (200 μL). Then, 2 μL of 0.1 mM Nile Red stock solution (in acetone) were added. The samples were vortexed briefly, sealed and incubated overnight at room temperature in the absence of light. Fluorescence spectra were recorded at room temperature using an excitation wavelength of 570 nm and monitoring emission between 600 and 740 nm, with excitation and emission slit widths both set at 5 nm.

Agarose gel electrophoresis assay. TAMRA-labeled oligonucleotides were diluted with TBS buffer to the final concentration of 5 μM and left at room temperature for two hours. Then, 20 μL of oligonucleotides were subjected to 1% agarose gel electrophoresis assay.

DLS assay of oligonucleotides. TAMRA-labeled oligonucleotides were diluted with TBS buffer to the final concentration of 10 μM and left at room temperature for two hours. Then, 100 μL of oligonucleotides were subjected to DLS assay.

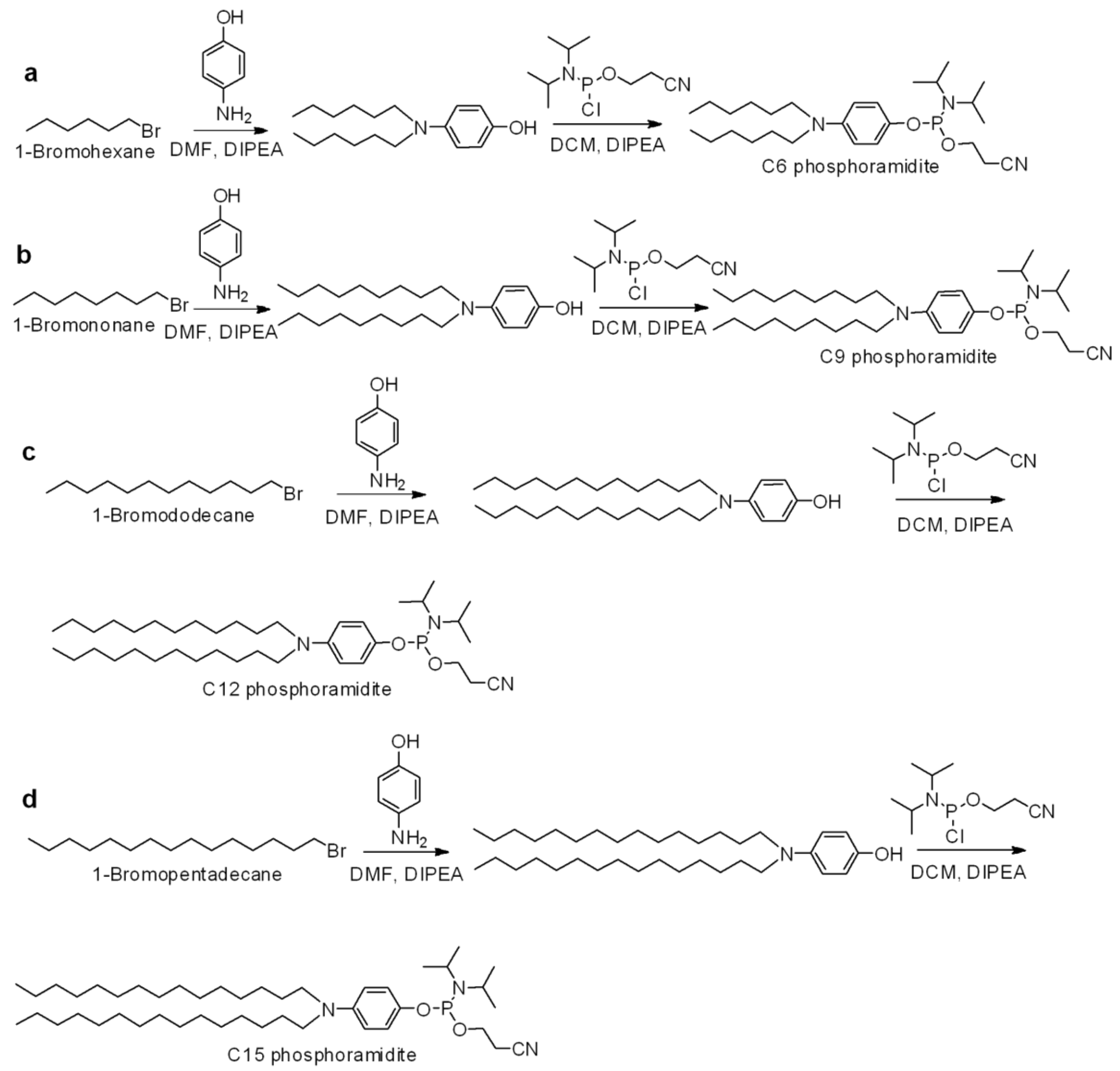

Supplementary Figure 1 Synthesis routes of C6, C9, C12 and C15 phosphoramidites. **a** Synthesis routes of C6 phosphoramidites. **b** Synthesis routes of C9 phosphoramidites. **c** Synthesis routes of C12 phosphoramidites. **d** Synthesis routes of C15 phosphoramidites.

Supplementary Table 1 DNA sequences designed in this work.

Name	Sequence (from 5’ to 3’)
DNA	TTT TTT TTT TTT TTT TTT TT
DNA-TAMRA	TTT TTT TTT TTT TTT TTT TT-TAMRA
C6-DNA	C6-TTT TTT TTT TTT TTT TTT TT
C6-DNA-TAMRA	C6-TTT TTT TTT TTT TTT TTT TT-TAMRA
C9-DNA	C9-TTT TTT TTT TTT TTT TTT TT
C9-DNA-TAMRA	C9-TTT TTT TTT TTT TTT TTT TT-TAMRA
C12-DNA	C12-TTT TTT TTT TTT TTT TTT TT
C12-DNA-TAMRA	C12-TTT TTT TTT TTT TTT TTT TT-TAMRA
C15-DNA	C15-TTT TTT TTT TTT TTT TTT TT
C15-DNA-TAMRA	C15-TTT TTT TTT TTT TTT TTT TT-TAMRA
DNA-lipid	Lipid-TTT TTT TTT TTT TTT TTT TT
DNA-lipid-TAMRA	Lipid-TTT TTT TTT TTT TTT TTT TT-TAMRA
DNA-lipid-P	P-lipid-TTT TTT TTT TTT TTT TTT TT
DNA-lipid-P-TAMRA	P-lipid-TTT TTT TTT TTT TTT TTT TT-TAMRA
Dabcyl-cDNA	Dabcyl-AAA AAA AAA AAA AAA AAA AA

Note: P indicates phosphate group; TAMRA indicates tetramethyl rhodamine group; Dabcyl indicates 4-(dimethylamino) azobenzene group; T indicates thymidine.

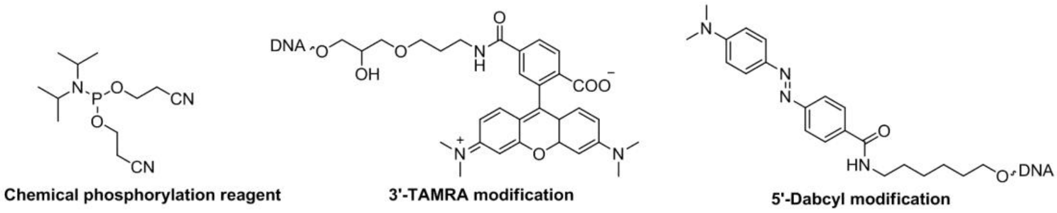

Supplementary Figure 2 Chemical structures of chemical compounds. Left: chemical phosphorylation reagent (bis(2-cyanoethyl)-N,N-diisopropyl phosphoramidite). Middle: 3’-TAMRA modification. Right: 5’-Dabcyl modification.

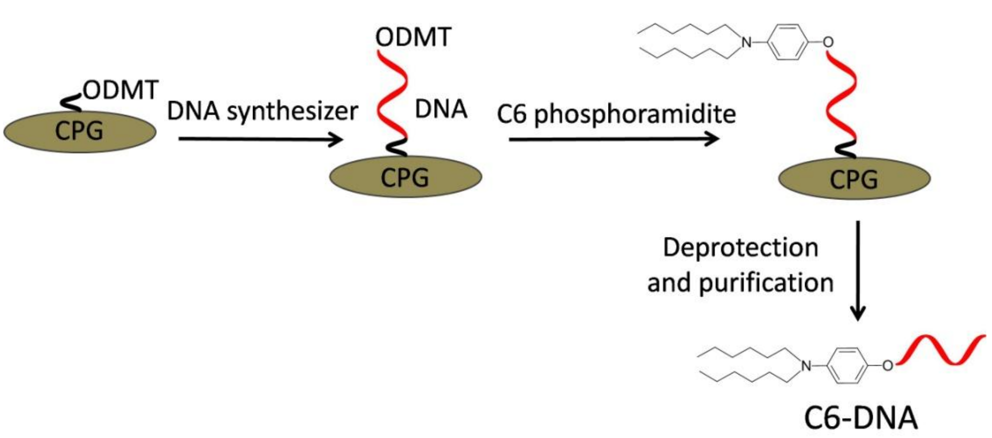

Supplementary Figure 3 DNA synthesis route of C6-DNA. The synthesis route of C9-DNA, C12-DNA and C15-DNA is the same as that of C6-DNA.

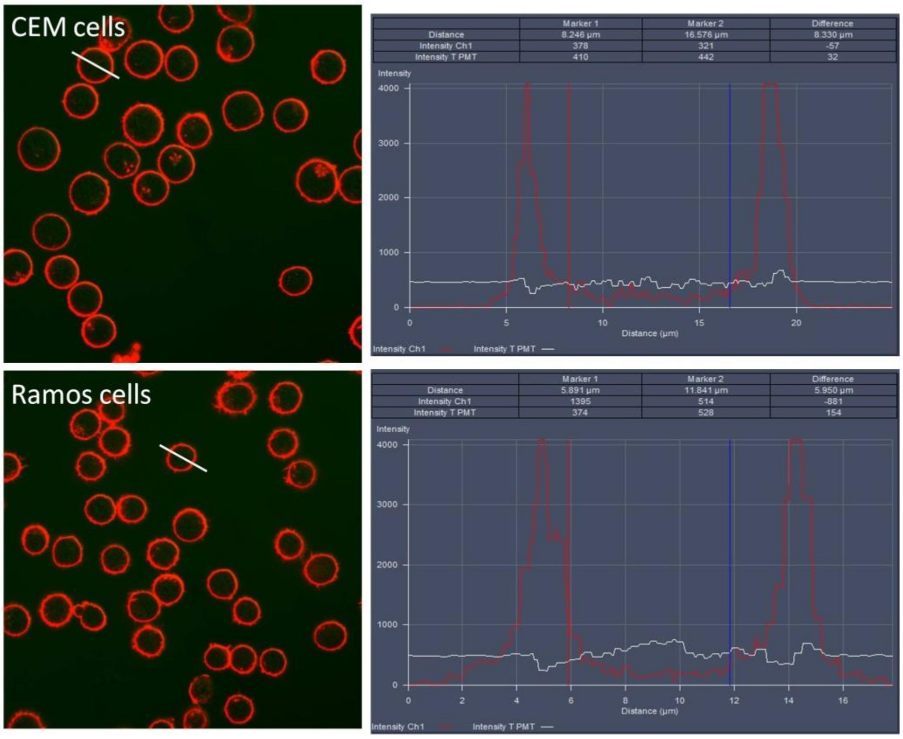

Supplementary Figure 4 Confocal fluorescence microscopy imaging of suspension culture cells (CEM and Ramos cells) treated with 1 μM TAMRA-labeled C15-DNA for one hour at room temperature. C15-DNA, a conventional lipid-conjugated oligonucleotide, cannot selectively anchor on either CEM or Ramos cell membranes. In other words, conventional lipid-conjugated oligonucleotides lack of selectivity among these cell membranes.

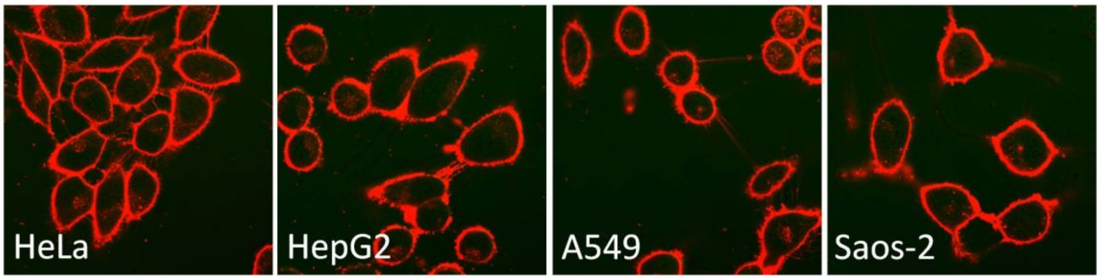

Supplementary Figure 5 Confocal fluorescence microscopy imaging of adherent culture cells (HeLa, HepG2, A549 and Saos-2 cells) treated with 1 μM TAMRA-labeled C15-DNA for one hour at room temperature. C15-DNA, a conventional lipid-conjugated oligonucleotide, shows poor selectivity among these cell membranes.

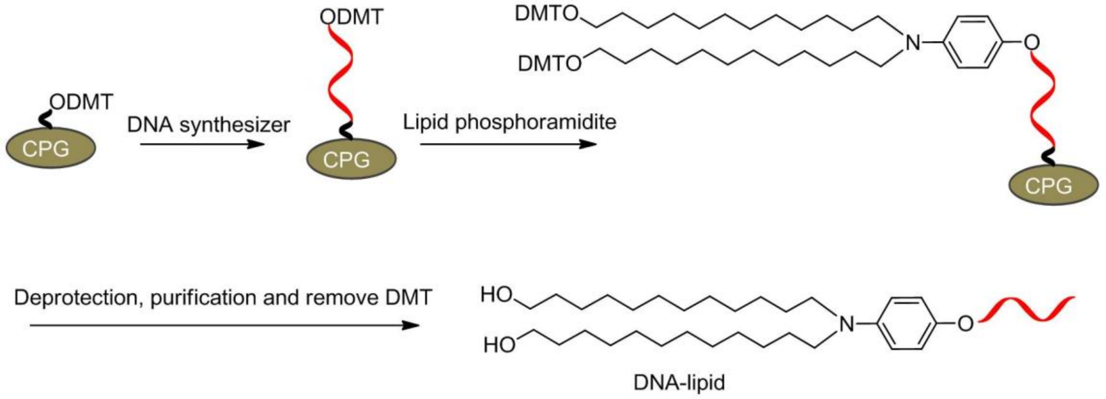

Supplementary Figure 6 DNA synthesis route of DNA-lipid.

Supplementary Table 2 HPLC purification program

Time/min	A (0.1 M TEAA)	B (acetonitrile)
0	95%	5%
4	95%	5%
4.01	90%	10%
40	5%	95%
50	5%	95%

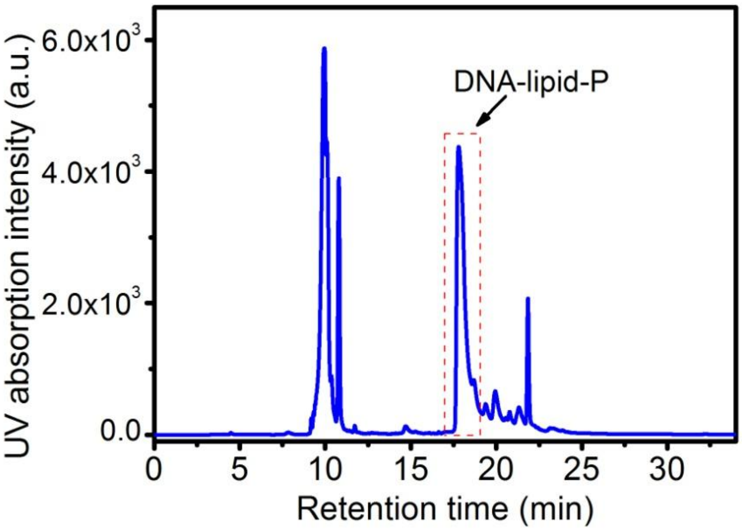

Supplementary Figure 7 Purification profile of DNA-lipid-P. DNA peak in red frame is DNA-lipid-P.

Supplementary Table 3 Mass spectrometry analysis of oligonucleotides.

Oligonucleotides	Calculated mass/Da	Observed mass/Da	Purity (estimated)
DNA	6022.0	6022.6 (Supplementary Fig. 21)	93.0%
C6-DNA	6361.4	6360.9 (Supplementary Fig. 22)	96.6%
C9-DNA	6445.3	6445.5 (Supplementary Fig. 23)	94.0%
C12-DNA	6529.7	6529.8 (Supplementary Fig. 24)	96.8%
C15-DNA	6613.9	6613.7 (Supplementary Fig. 25)	98.6%
DNA-lipid	6562.6	6562.6 (Supplementary Fig. 26)	97.3%
DNA-lipid-P	6721.7	6721.6 (Supplementary Fig. 27)	99.8%

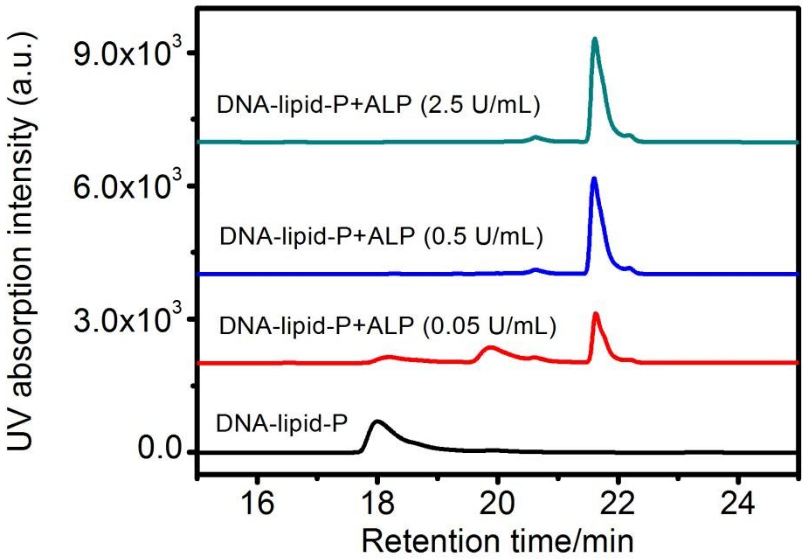

Supplementary Figure 8 HPLC chromatograms of DNA-lipid-P (black line) and DNA-lipid-P treated with different concentration of ALP. DNA peak in 18.0 minutes is DNA-lipid-P. DNA peak in 21.8 minutes is the product after dephosphorylation.

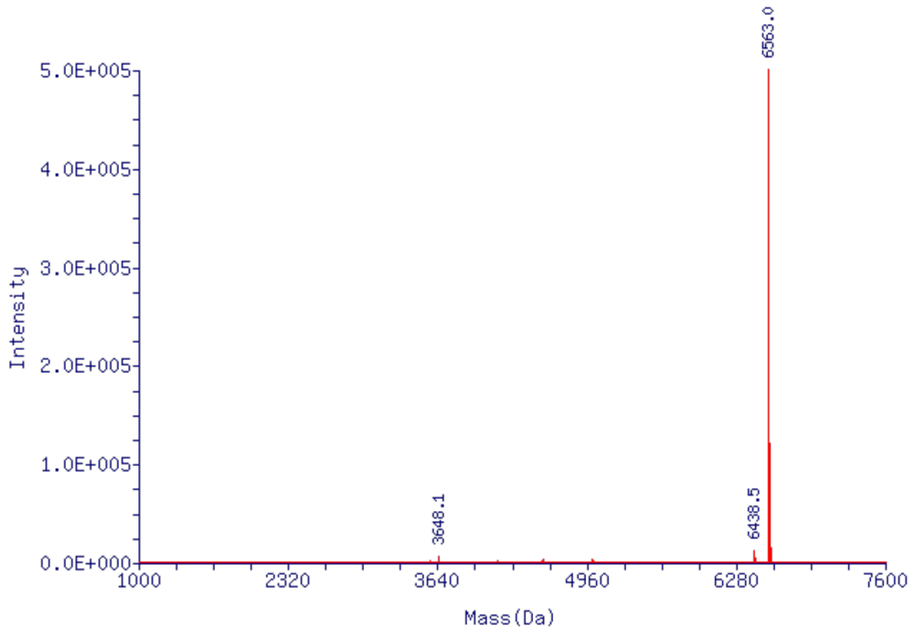

Supplementary Figure 9 Mass spectrometry analysis of the dephosphorylation product of DNA-lipid-P treated with ALP (0.5 U/mL). Calculated molecular weight is 6562.6 Da, and observed molecular weight is 6563.0 Da (the estimated purity is 96.7%), indicating that DNA-lipid-P was converted to DNA-lipid.

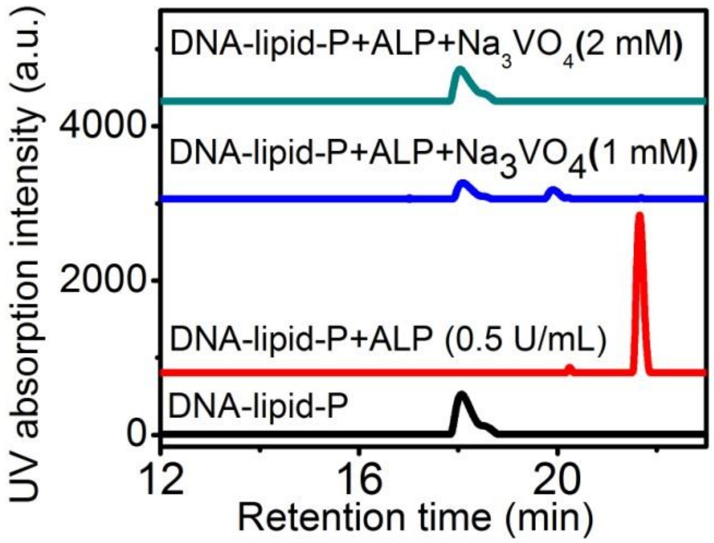

Supplementary Figure 10 HPLC chromatograms of DNA-lipid-P (black line), DNA-lipid-P treated with ALP (0.5 U/mL) (red line) and DNA-lipid-P treated with ALP (0.5 U/mL) in Na_3VO_4-containing (1 mM or 2 mM) buffer solution (blue and green lines).

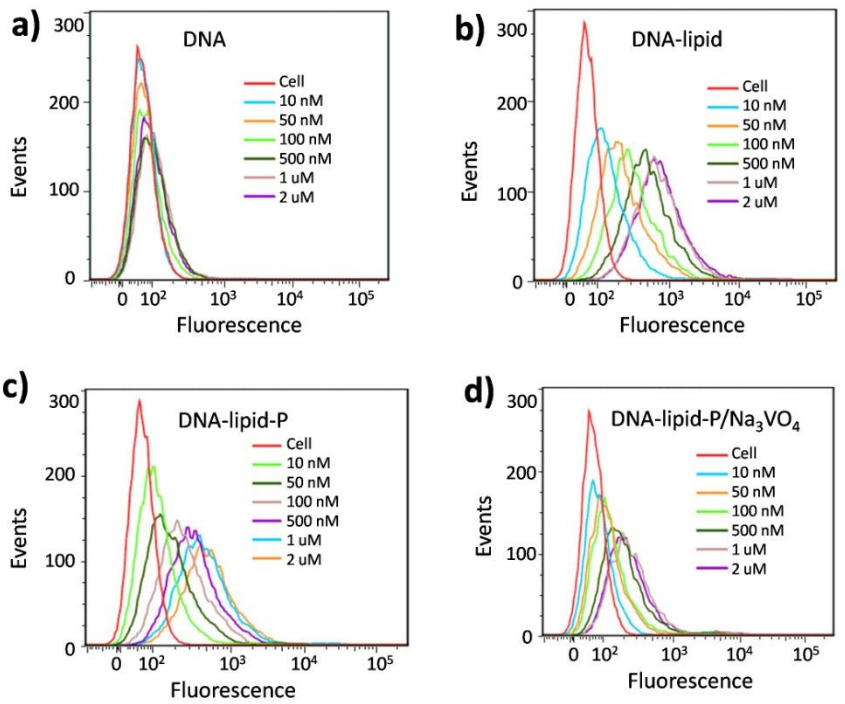

Supplementary Figure 11 Binding affinity analysis of oligonucleotides to HepG2 cells. **a** Flow cytometry assay of HepG2 cells treated with TAMRA-labeled DNA in TBS buffer solution for one hour at 37 °C. **b** Flow cytometry assay of HepG2 cells treated with TAMRA-labeled DNA-lipid in TBS buffer solution for one hour at 37 °C. **c** Flow cytometry assay of HepG2 cells treated with TAMRA-labeled DNA-lipid-P in TBS buffer solution for one hour at 37 °C. **d** Flow cytometry assay of HepG2 cells treated with TAMRA-labeled DNA-lipid-P+Na_3VO_4 (2 mM) in TBS buffer solution for one hour at 37 °C. The fluorescence was collected in PE channel.

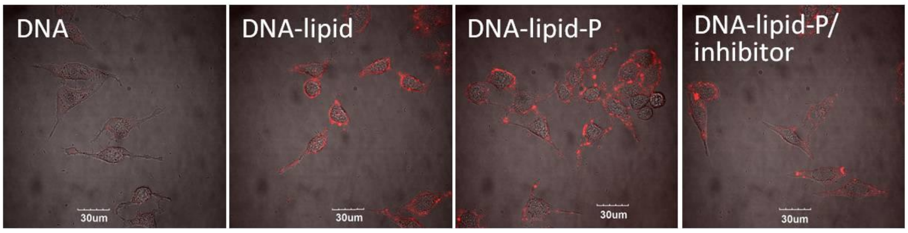

Supplementary Figure 12 Confocal fluorescence microscopy imaging of HepG2 cells treated with 1 μM TAMRA-labeled DNA, DNA-lipid, DNA-lipid-P or DNA-lipid-P+Na_3VO_4 in TBS buffer for two hours at 37 °C. Most fluorescence signals were located on the cell membrane. Scale bar is 30 μm.

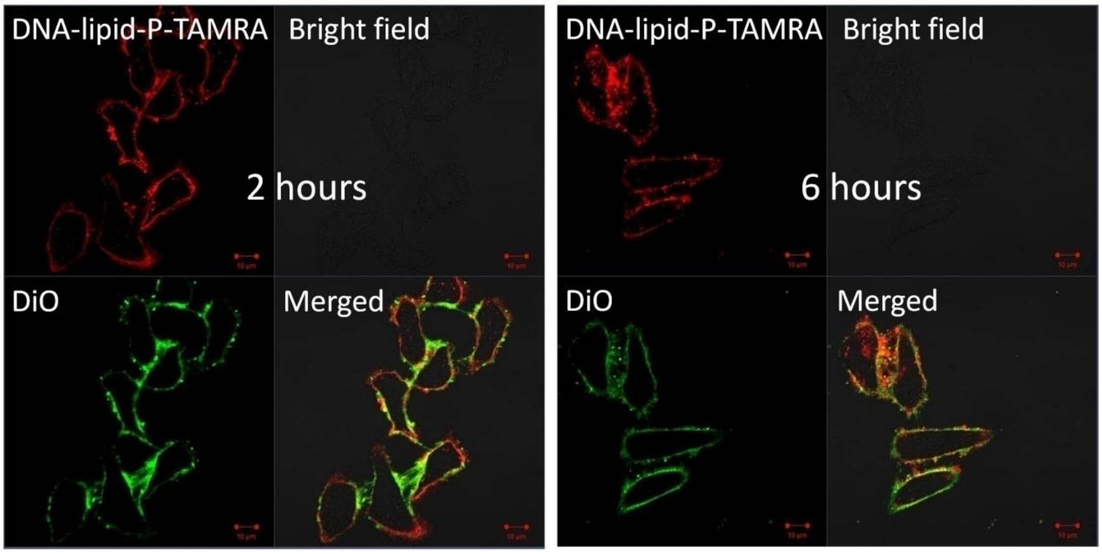

Supplementary Figure 13 Confocal fluorescence microscopy imaging of HepG2 cells treated with 1 μM TAMRA-labeled DNA-lipid-P in TBS buffer for two (left) and six (right) hours at 37 °C. The commercial cell membrane probe (DiO) was incubated with cells for 15 minutes at 37 °C. Scale bar is 10 μm.

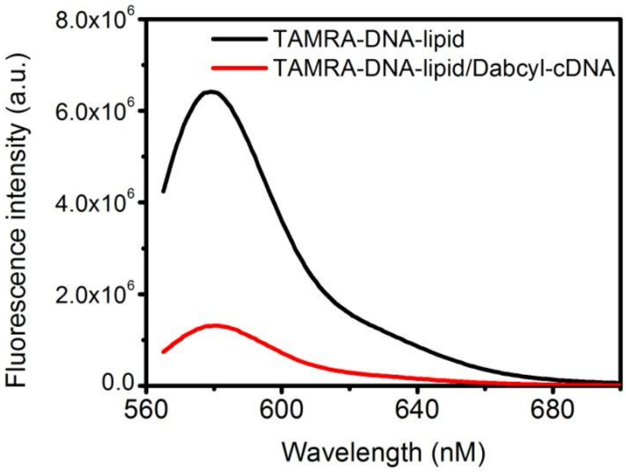

Supplementary Figure 14 Fluorescence spectra of TAMRA-labeled DNA-lipid before (black line) and after (red line) hybridization with Dabcyl-cDNA. The obvious decline of fluorescence intensity indicates the effective hybridization between DNA-lipid and cDNA.

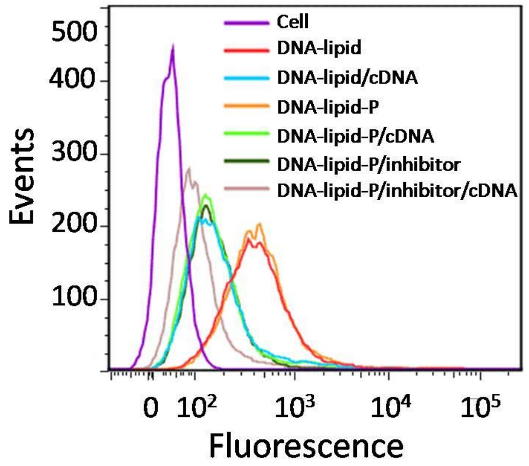

Supplementary Figure 15 Flow cytometry assay of HepG2 cells treated with 1 μM TAMRA-labeled DNA-lipid, DNA-lipid-P or DNA-lipid-P+Na_3VO_4 before or after hybridization with Dabcyl-cDNA. The fluorescence was collected in PE channel.

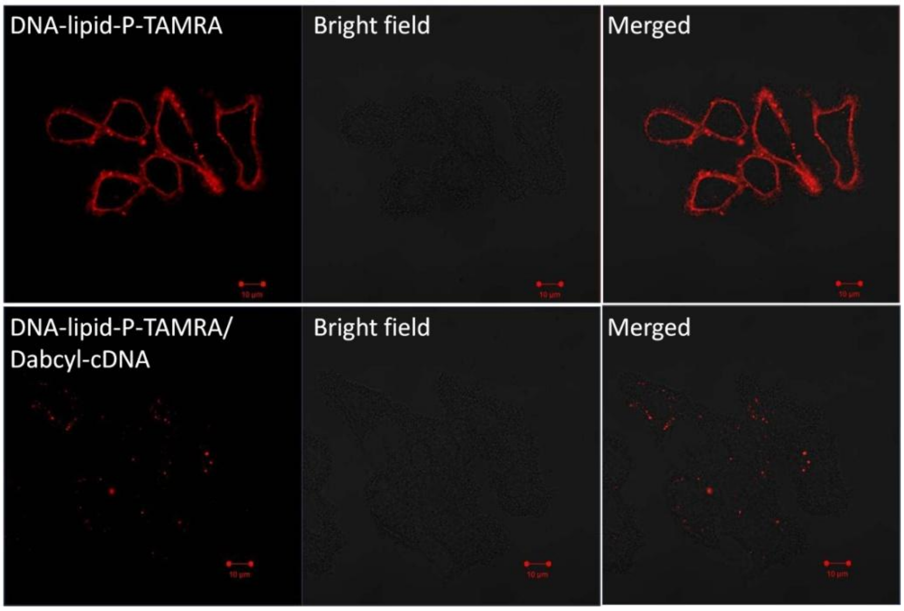

Supplementary Figure 16 Confocal fluorescence microscopy imaging of HepG2 cells treated with 1 μM TAMRA-labeled DNA-lipid-P before and after hybridization with Dabcyl-cDNA. HepG2 cells were incubated with DNA-lipid-P-TAMRA for one hour at 37 °C. When the incubation was completed, cells were washed twice with TBS buffer. Then, 1 μM Dabcyl-cDNA in TBS buffer was added for additional ten minutes. Cells were washed twice with TBS buffer and subject to imaging assay. Scale bar is 10 μm.

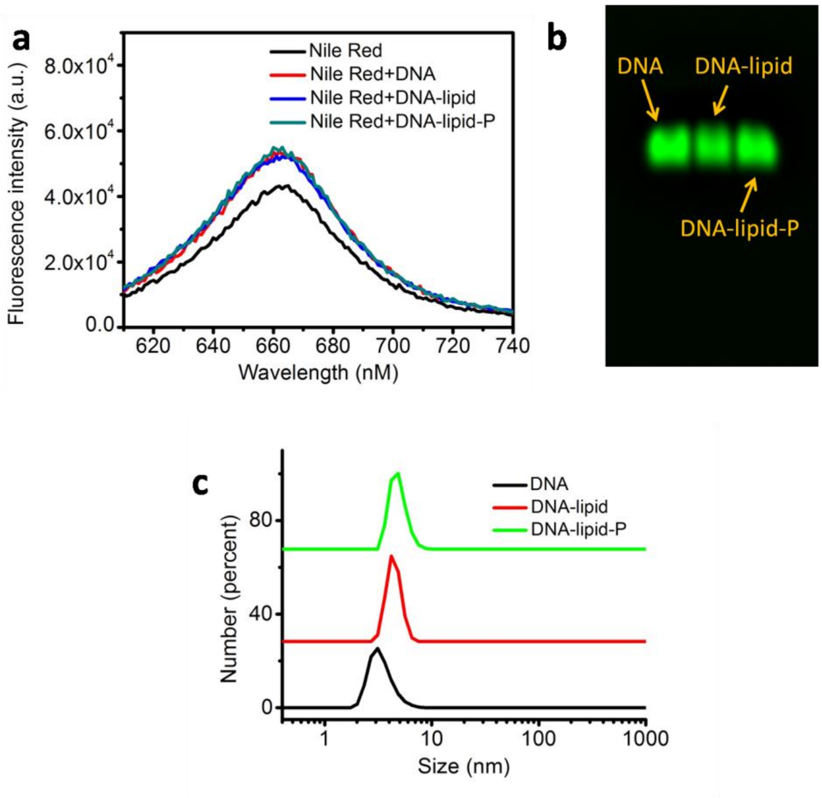

Supplementary Figure 17 Characterizations of dispersion state of oligonucleotides. **a** Fluorescence spectra of Nile Red-encapsulated DNA, DNA-lipid or DNA-lipid-P in TBS buffer solution. **b** 1% Agarose gel electrophoresis analysis of 5 µM TAMRA-labeled DNA, DNA-lipid or DNA-lipid-P. **c** Dynamic light scattering (DLS) analysis of 10 µM TAMRA-labeled DNA, DNA-lipid or DNA-lipid-P in buffer solution. No obvious fluorescence enhancement was observed in fluorescence spectra (a); TAMRA-labeled DNA, DNA-lipid and DNA-lipid-P have similar migration rates in agarose gel (b); and no obvious difference in size was observed between TAMRA-labeled DNA (3.1 nm), DNA-lipid (4.2 nm) and DNA-lipid-P (4.8 nm). This cumulative evidence shows that neither DNA-lipid nor DNA-lipid-P has appreciable aggregation into micellar nanoparticles. In other words, both DNA-lipid and DNA-lipid-P interact with cell membrane as monomer structure, not aggregated state.

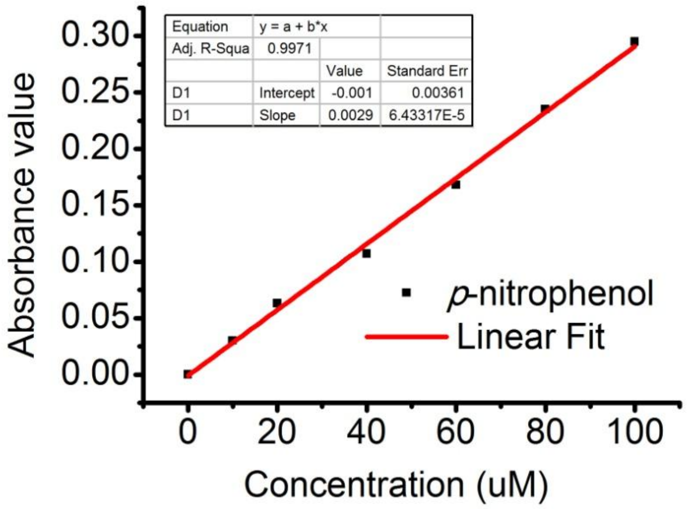

Supplementary Figure 18 Fitted function of the absorbance value of *p*-nitrophenol at 410 nm to the concentration of *p*-nitrophenol.

Supplementary Table 4 The measured absorbance of *p*-nitrophenyl phosphate in HepG2 and U-2 OS cell lysis buffer.

Assay	Blank	HepG2	U-2 OS
R1	0.062	1.050	0.061
R2	0.062	1.094	0.065
R3	0.060	1.154	0.065

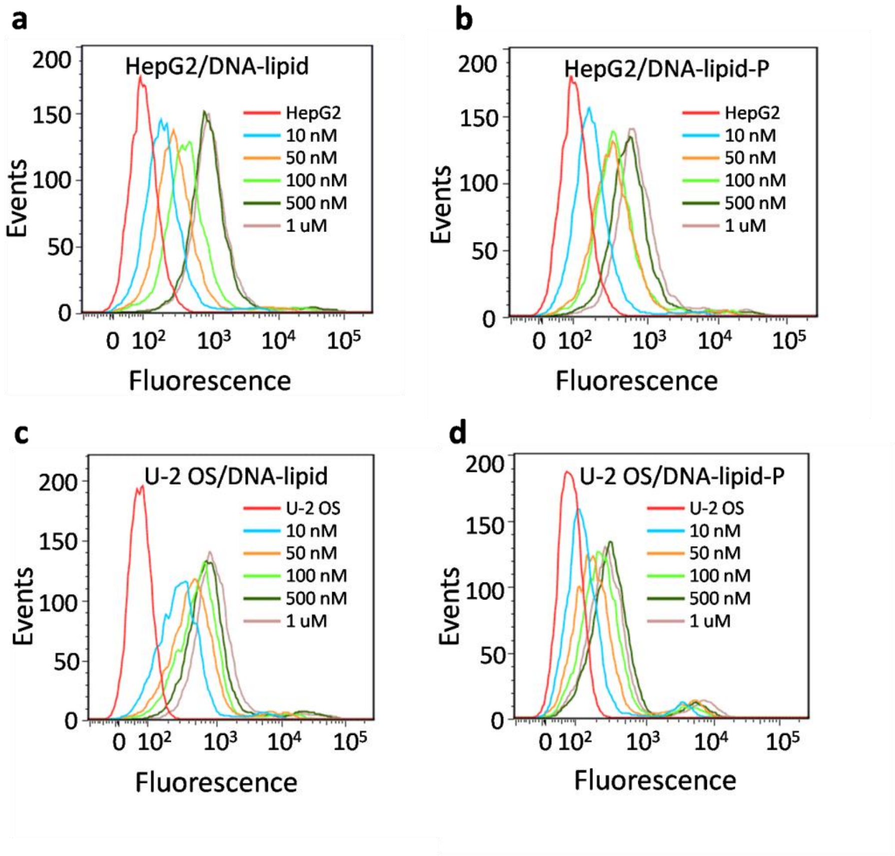

Supplementary Figure 19 Binding affinity assays. **a** Flow cytometry of HepG2 cells treated with TAMRA-labeled DNA-lipid in TBS buffer solution for one hour at 37 °C. **b** Flow cytometry of HepG2 cells treated with TAMRA-labeled DNA-lipid-P in TBS buffer solution for one hour at 37 °C. **c** Flow cytometry of U-2 OS cells treated with TAMRA-labeled DNA-lipid in TBS buffer solution for one hour at 37 °C. **d** Flow cytometry of U-2 OS cells treated with TAMRA-labeled DNA-lipid-P in TBS buffer solution for one hour at 37 °C. The fluorescence was collected in PE channel.

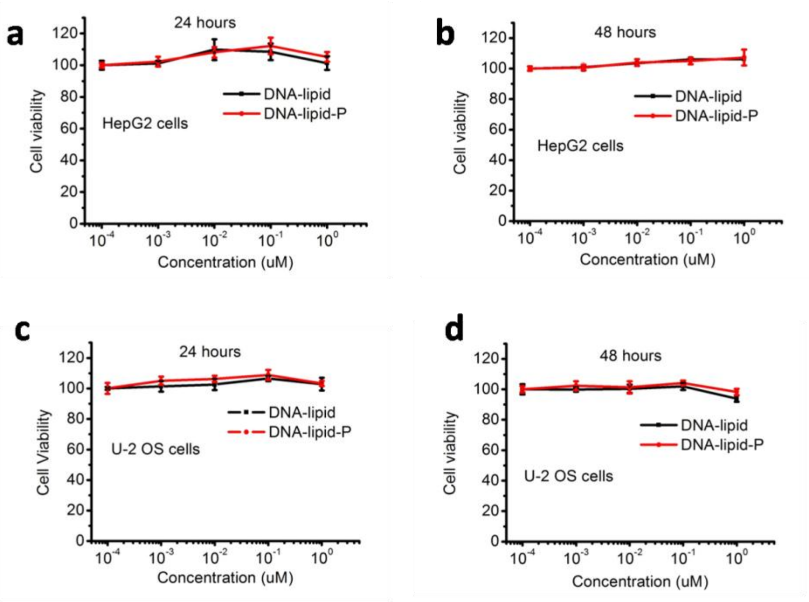

Supplementary Figure 20 Cell viability assays. **a** Cell viability assays of HepG2 cells treated with DNA-lipid or DNA-lipid-P for 24 hours. **b** Cell viability assays of HepG2 cells treated with DNA-lipid or DNA-lipid-P for 48 hours. **c** Cell viability assays of U-2 OS cells treated with DNA-lipid or DNA-lipid-P for 24 hours. **d** Cell viability assays of U-2 OS cells treated with DNA-lipid or DNA-lipid-P for 48 hours.

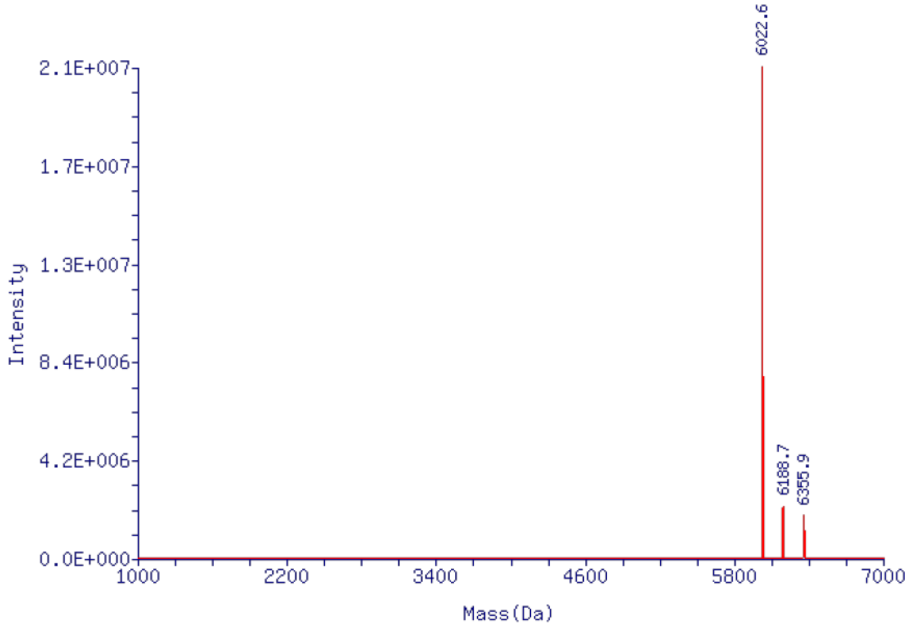

Supplementary Figure 21 Mass spectrometry analysis of DNA. Calculated molecular weight is 6022.0, and observed molecular weight is 6022.6. The estimated purity is 93.0%.

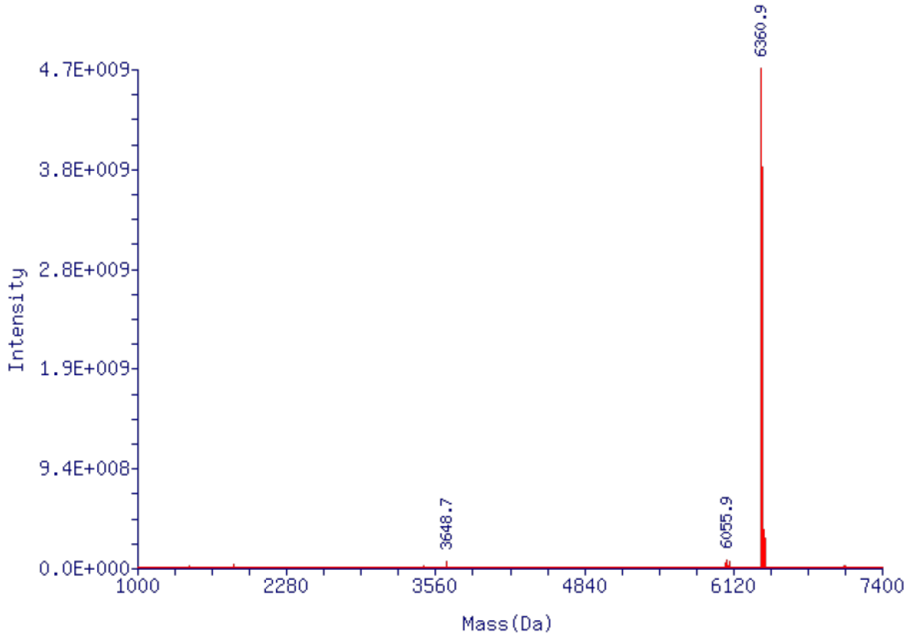

Supplementary Figure 22 Mass spectrometry analysis of C6-DNA. Calculated molecular weight is 6361.4, and observed molecular weight is 6360.9. The estimated purity is 96.6%.

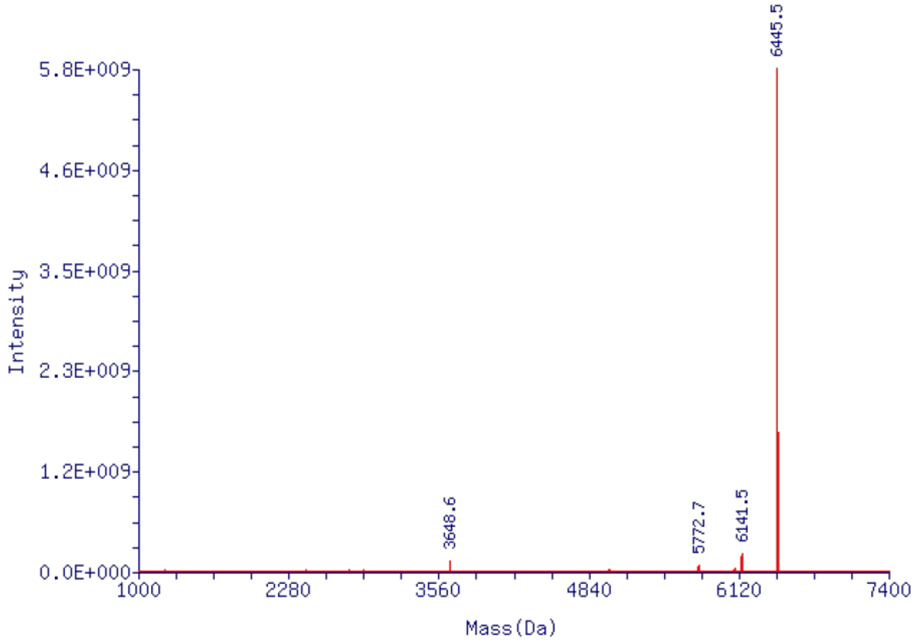

Supplementary Figure 23 Mass spectrometry analysis of C9-DNA. Calculated molecular weight is 6445.3, and observed molecular weight is 6445.5. The estimated purity is 94.0%.

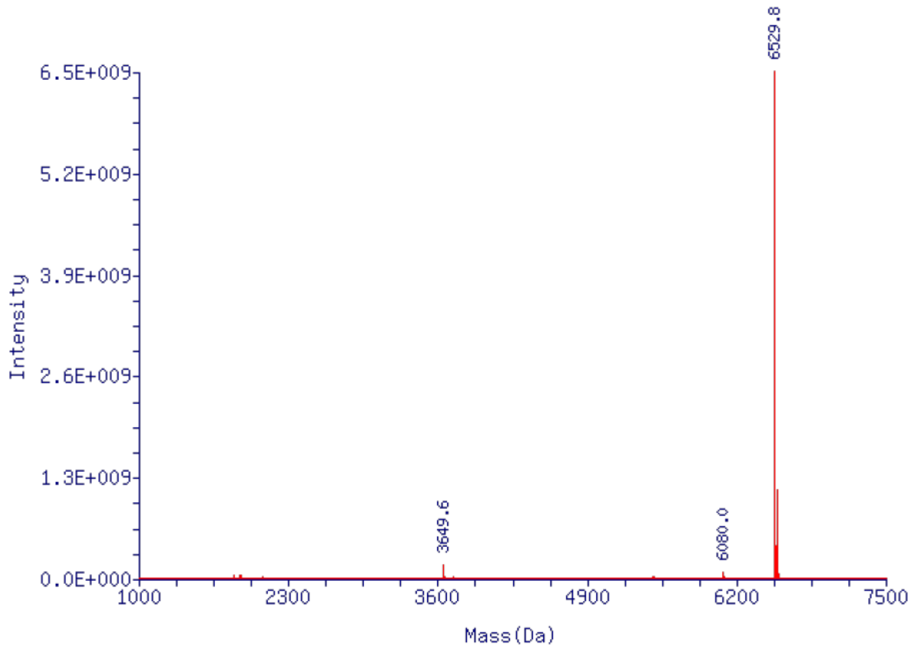

Supplementary Figure 24 Mass spectrometry analysis of C12-DNA. Calculated molecular weight is 6529.7, and observed molecular weight is 6529.8. The estimated purity is 96.8%.

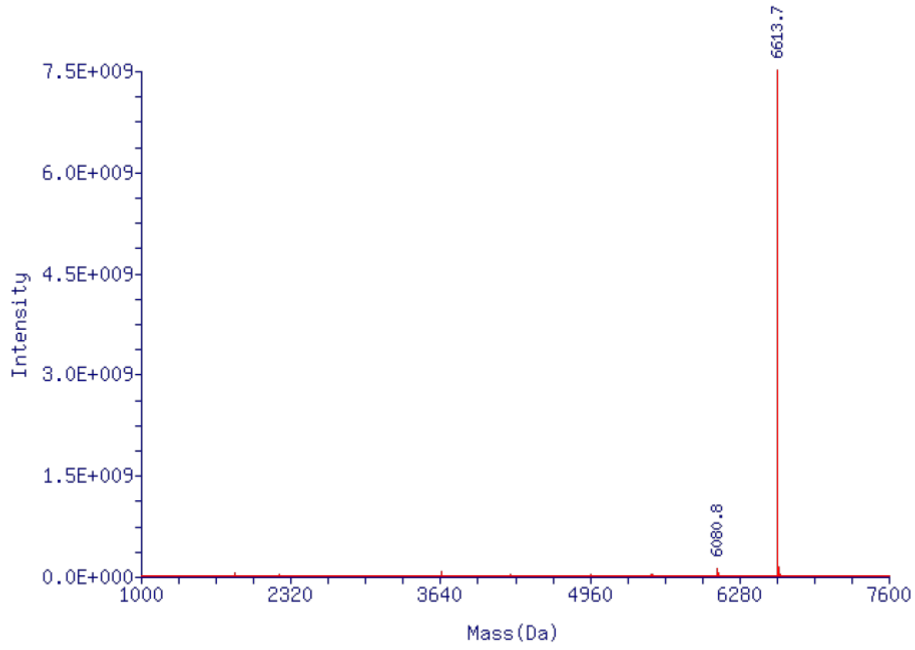

Supplementary Figure 25 Mass spectrometry analysis of C15-DNA. Calculated molecular weight is 6613.9, and observed molecular weight is 6613.7. The estimated purity is 98.6%.

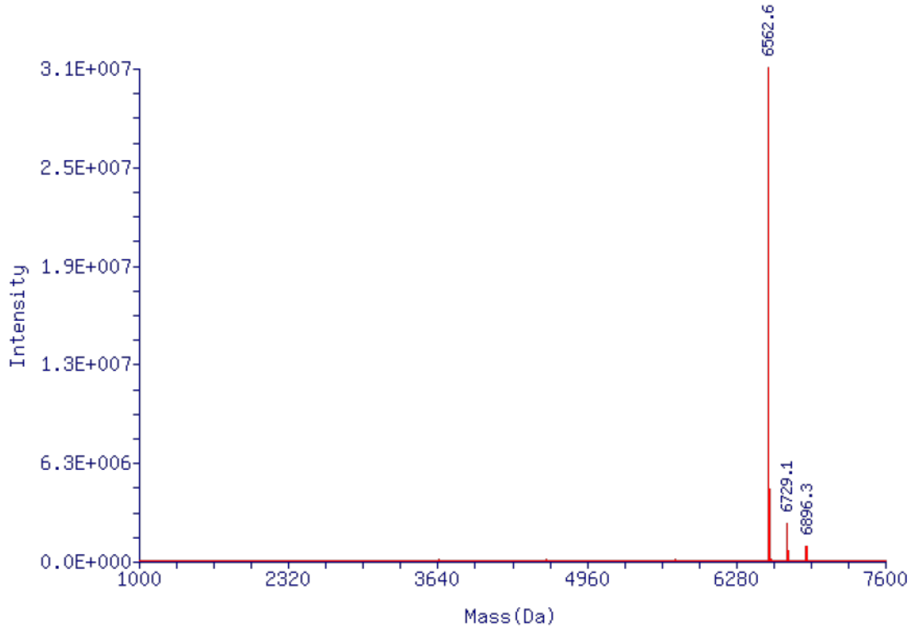

Supplementary Figure 26 Mass spectrometry analysis of DNA-lipid. Calculated molecular weight is 6562.6, and observed molecular weight is 6562.6. The estimated purity is 97.3%.

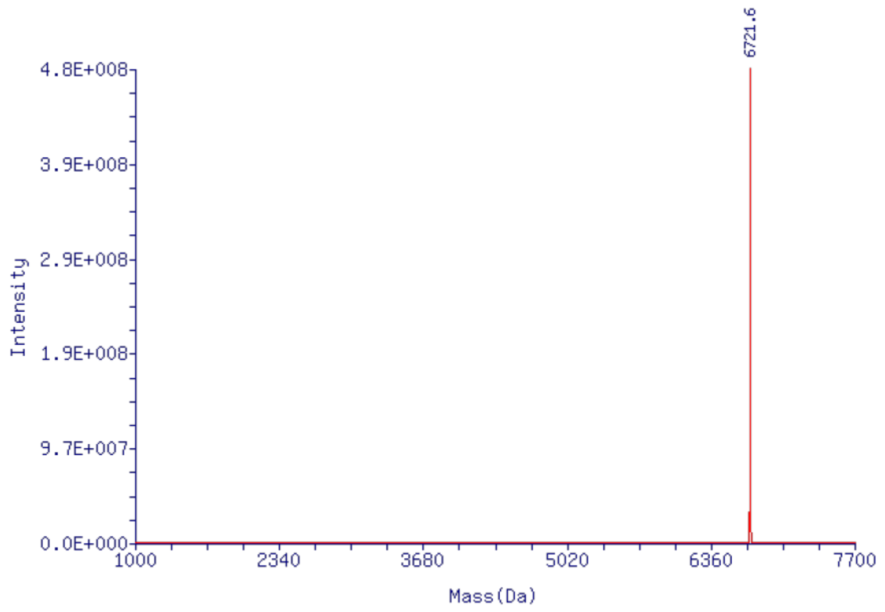

Supplementary Figure 27 Mass spectrometry analysis of DNA-lipid-P. Calculated molecular weight is 6721.7, and observed molecular weight is 6721.6. The estimated purity is 99.8%.

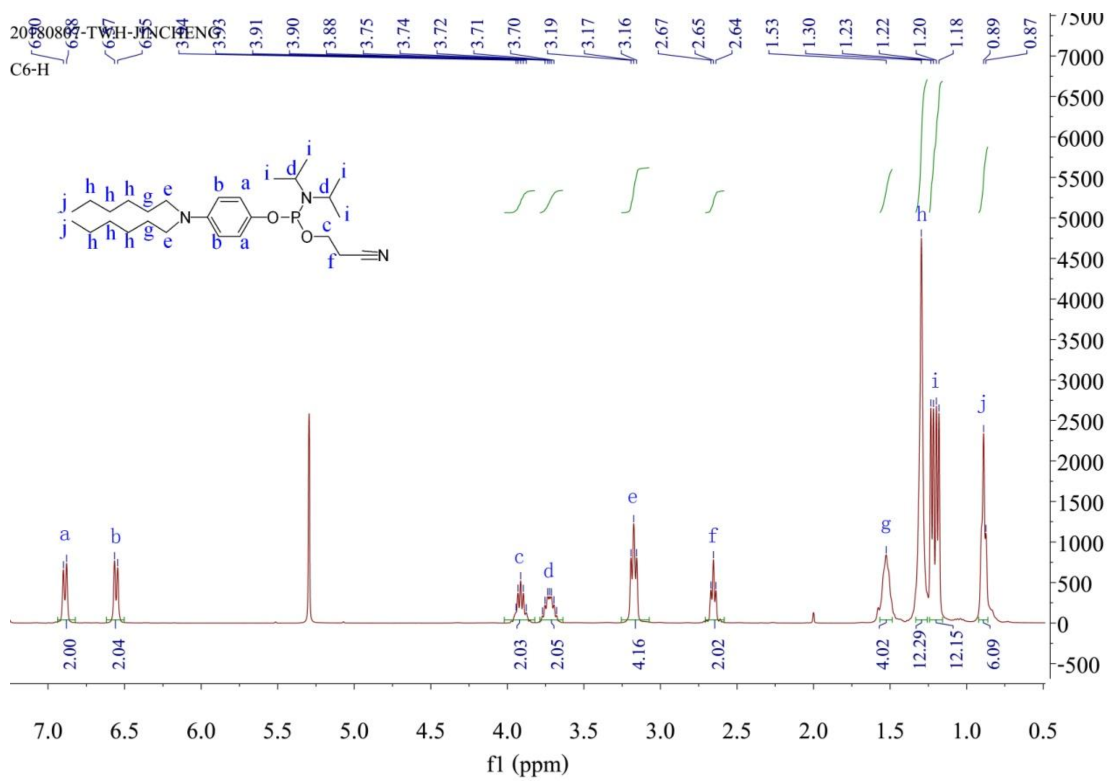

Supplementary Figure 28 ^{1}H NMR spectra of C6 phosphoramidite. ^{1}H NMR peak at 5.30 (s) is the peak of residual dichloromethane.

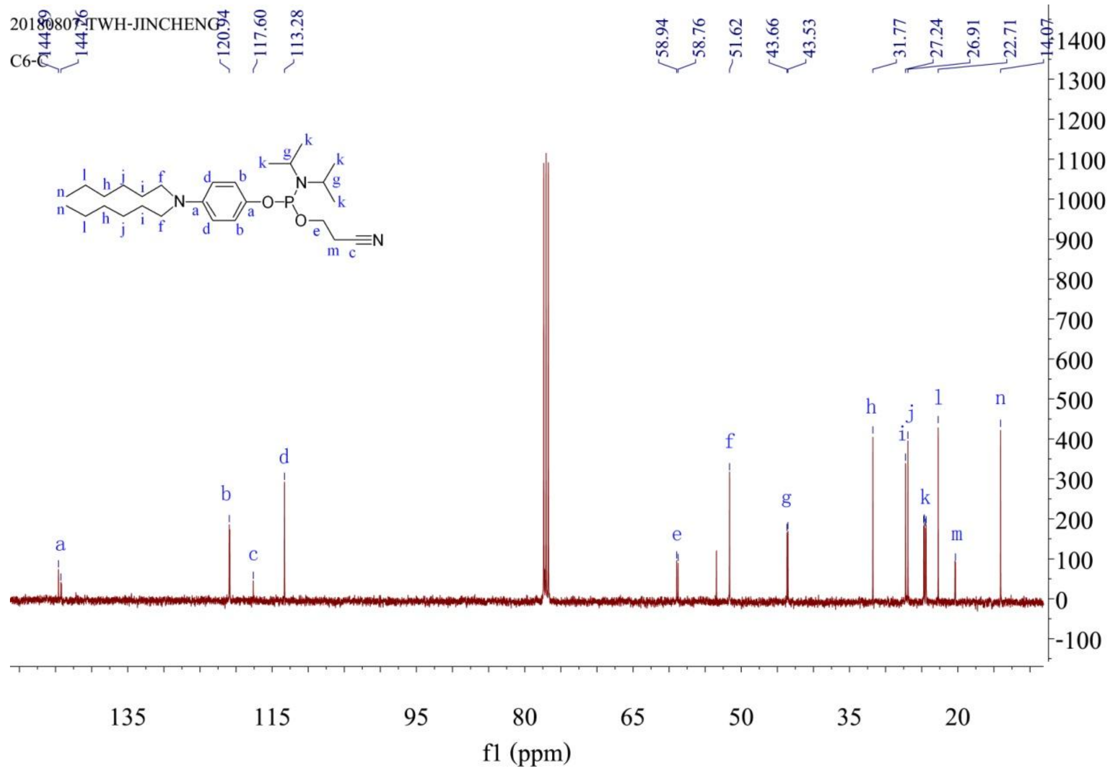

Supplementary Figure 29 ^{13}C NMR spectra of C6 phosphoramidite.

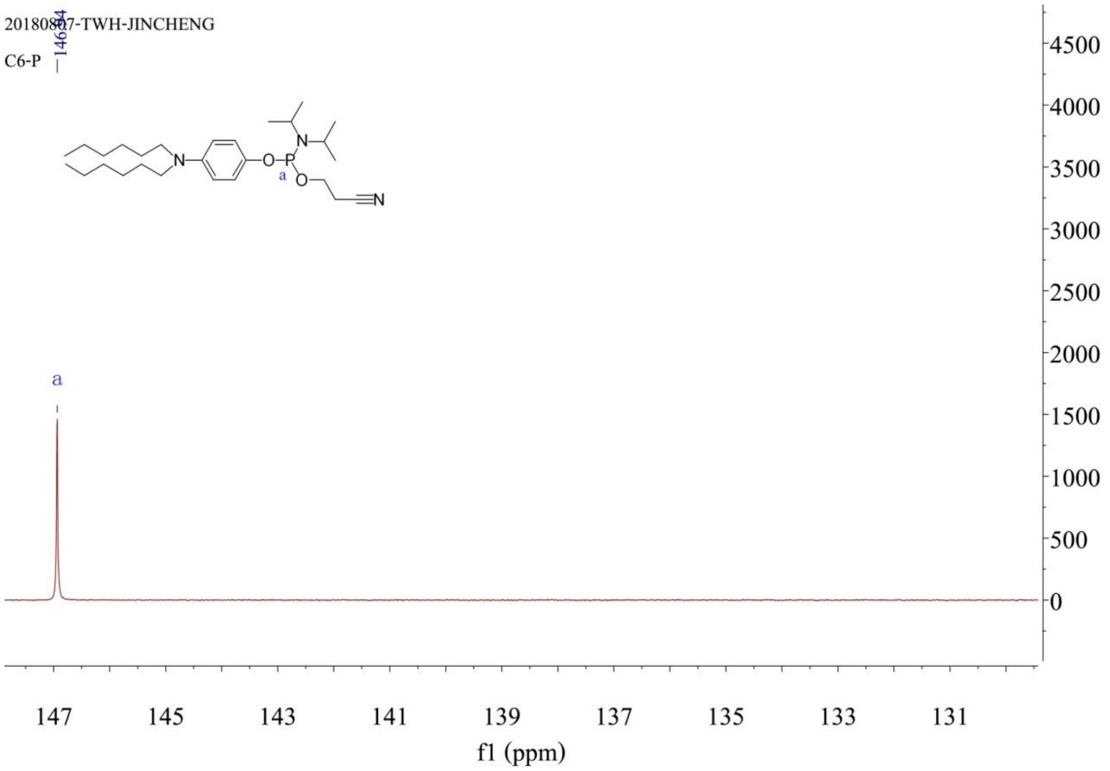

Supplementary Figure 30 ^{31}P NMR spectra of C6 phosphoramidite.

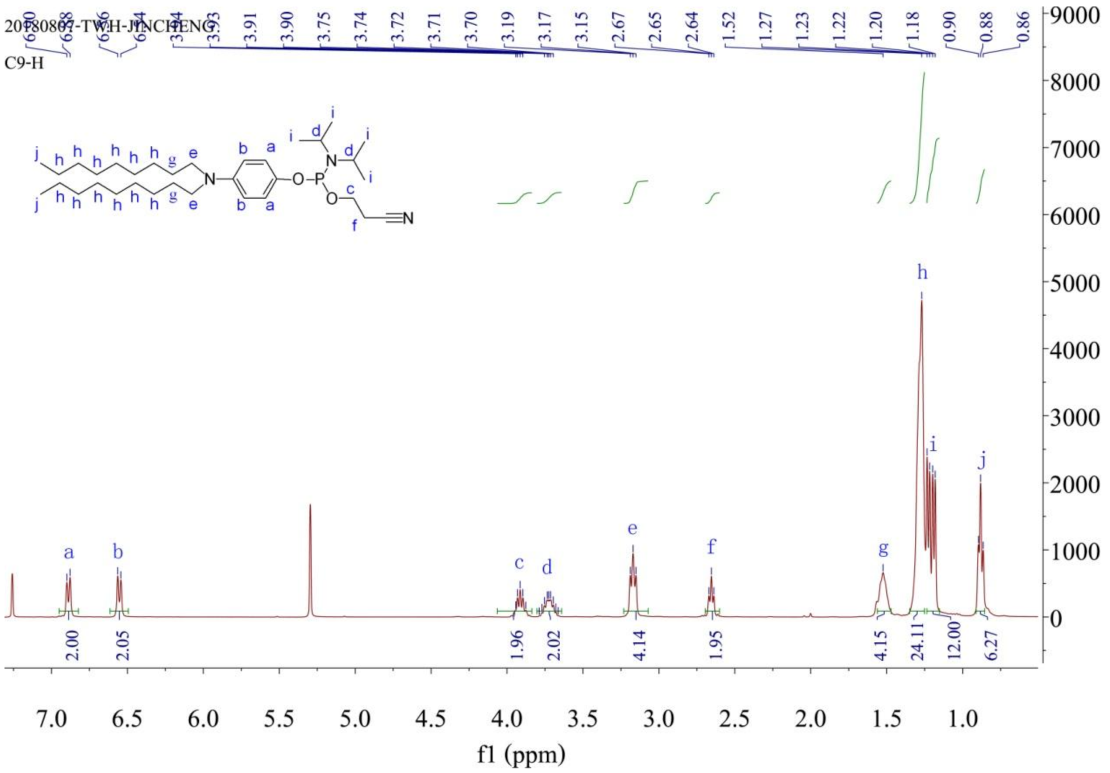

Supplementary Figure 31 ^{1}H NMR spectra of C9 phosphoramidite. ^{1}H NMR peak at 5.30 (s) is the peak of residual dichloromethane.

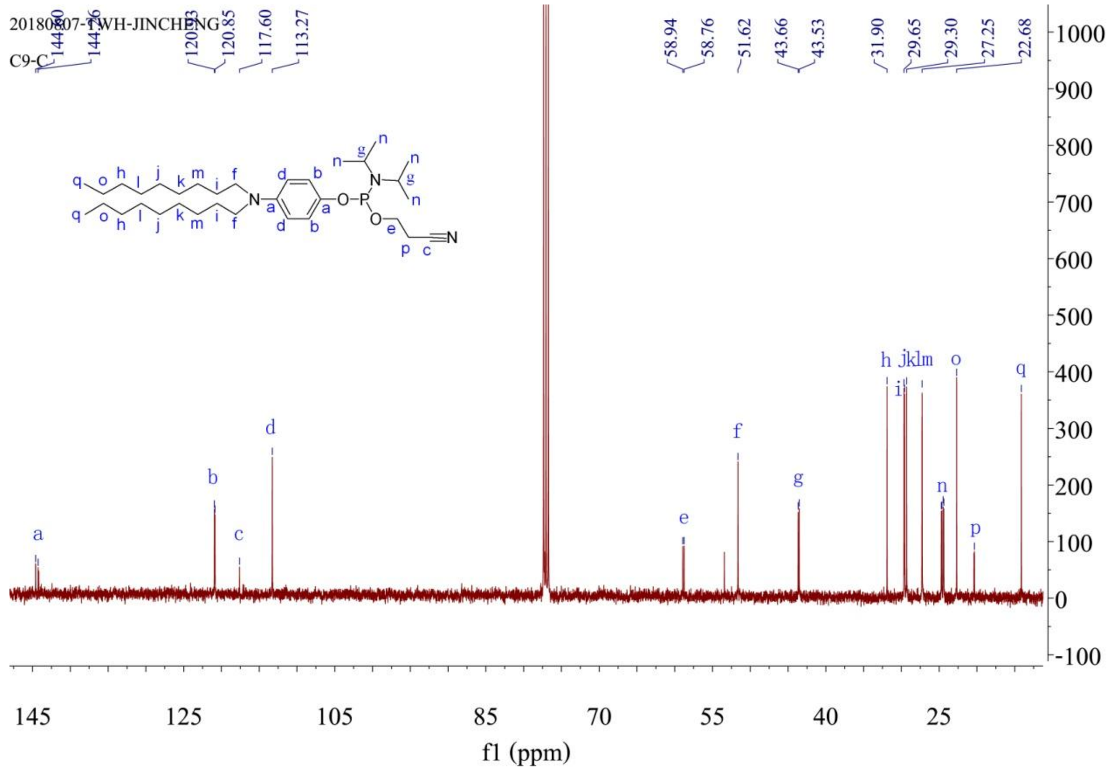

Supplementary Figure 32 ^{13}C NMR spectra of C9 phosphoramidite.

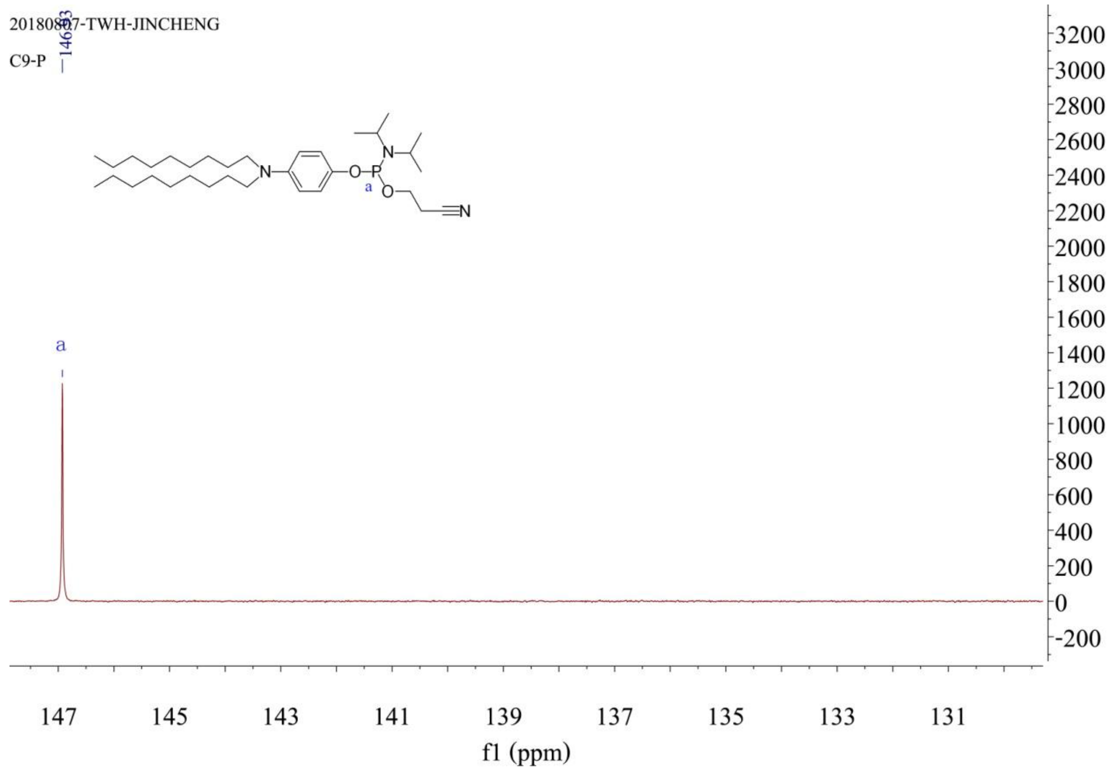

Supplementary Figure 33 ^{31}P NMR spectra of C9 phosphoramidite.

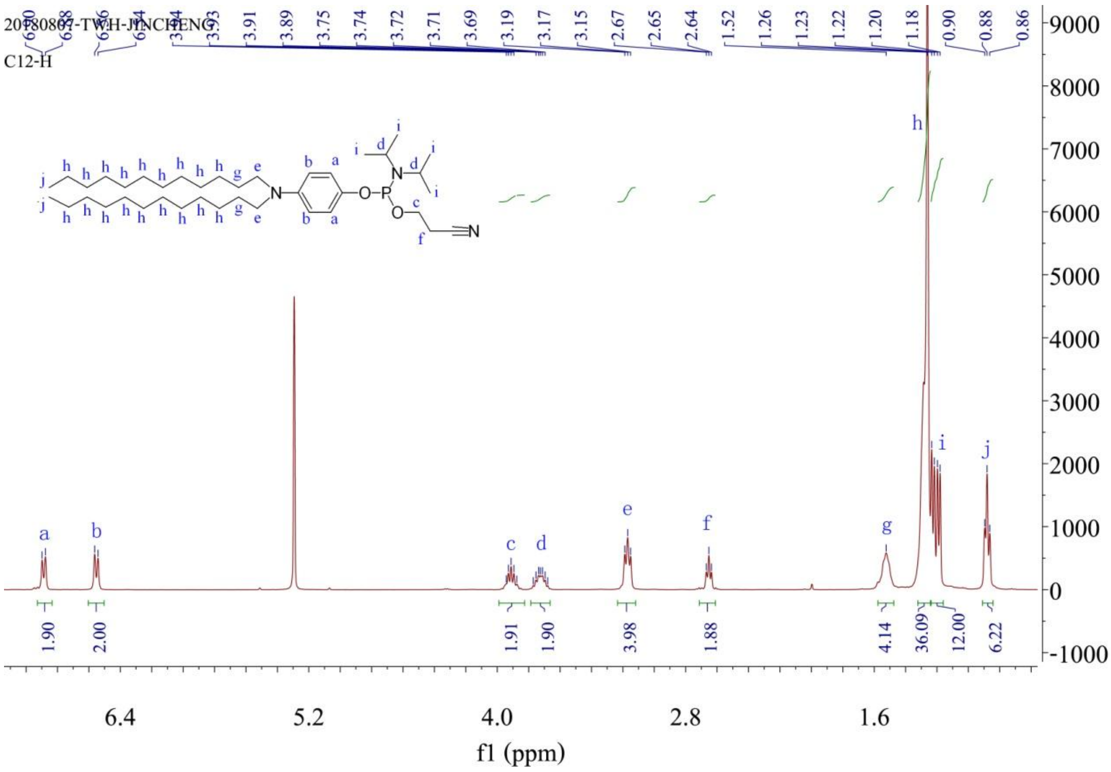

Supplementary Figure 34 ^{1}H NMR spectra of C12 phosphoramidite. ^{1}H NMR peak at 5.30 (s) is the peak of residual dichloromethane.

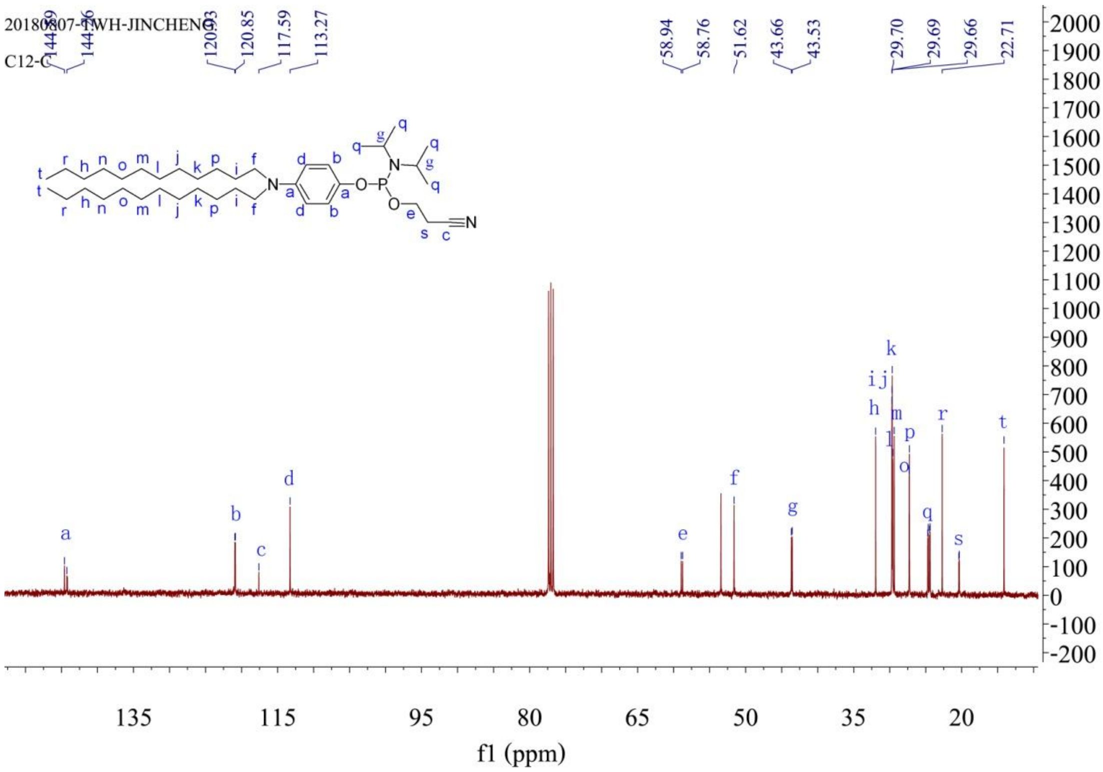

Supplementary Figure 35 ^{13}C NMR spectra of C12 phosphoramidite.

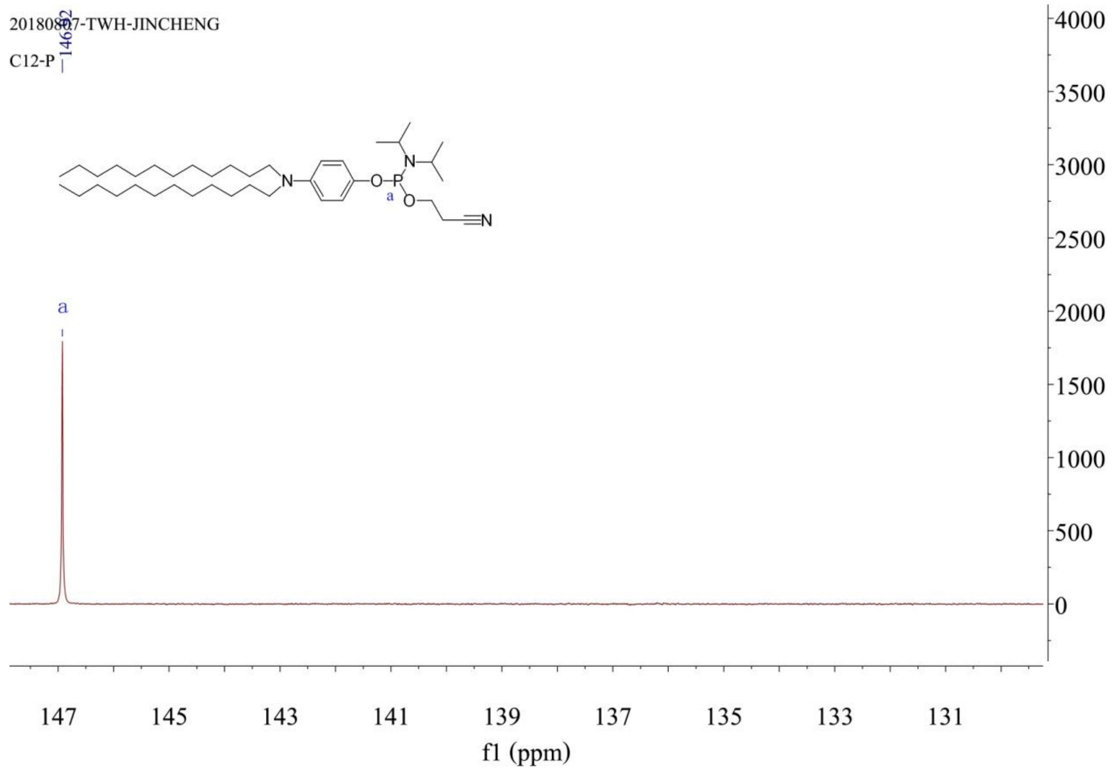

Supplementary Figure 36 ^{31}P NMR spectra of C12 phosphoramidite.

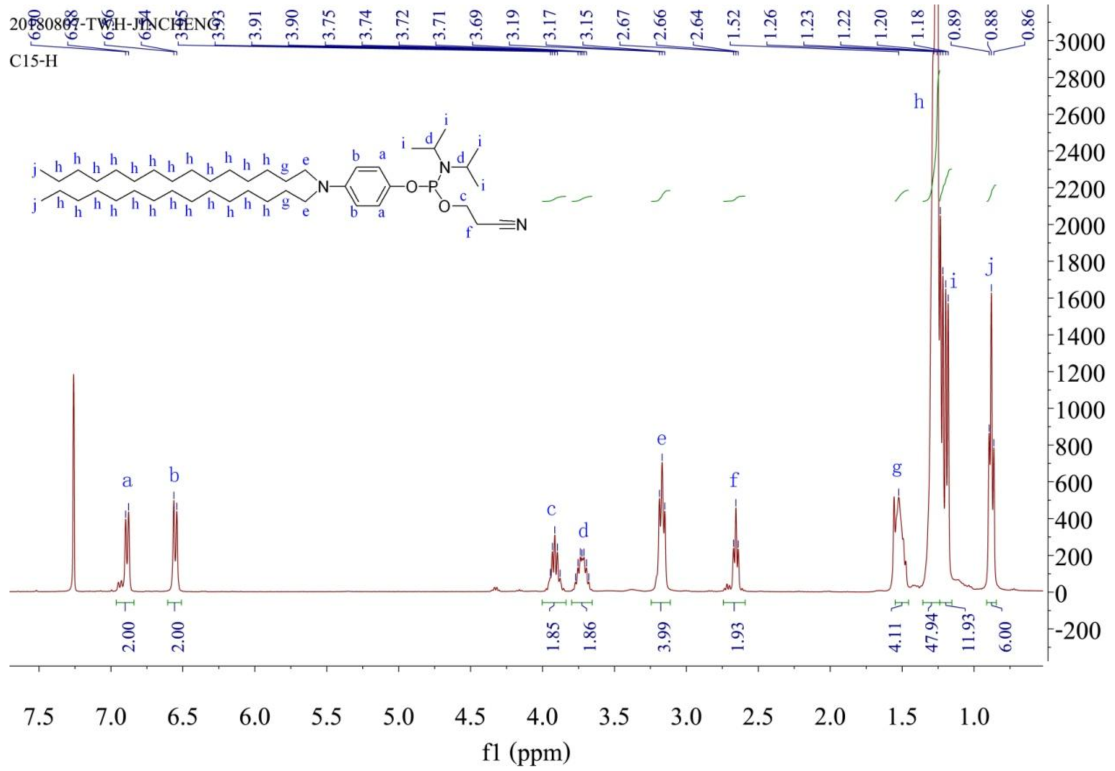

Supplementary Figure 37 ^{1}H NMR spectra of C15 phosphoramidite.

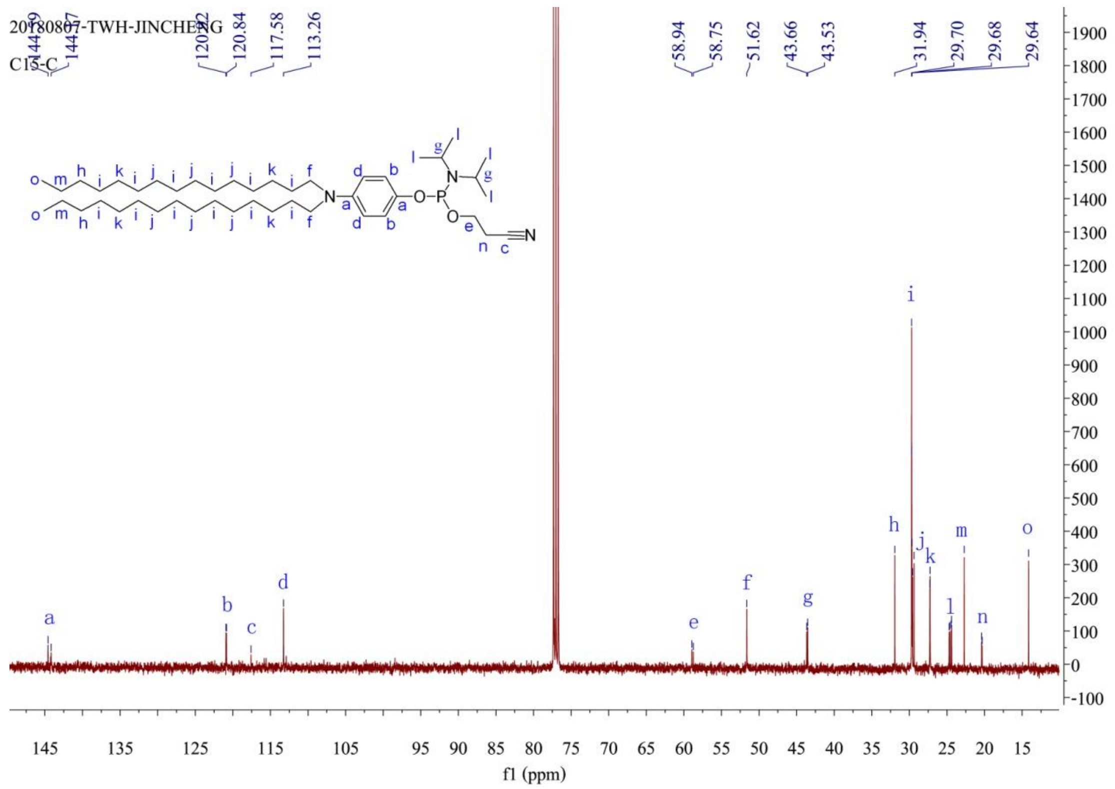

Supplementary Figure 38 ^{13}C NMR spectra of C15 phosphoramidite.

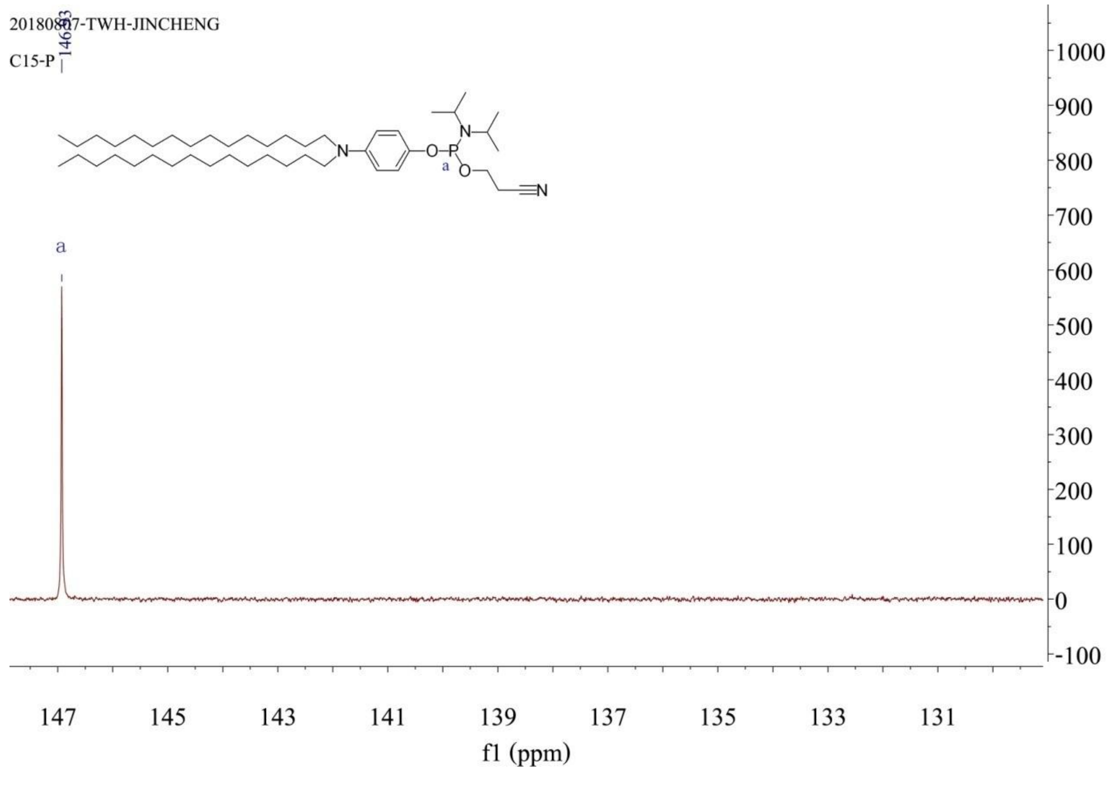

Supplementary Figure 39 ^{31}P NMR spectra of C15 phosphoramidite.

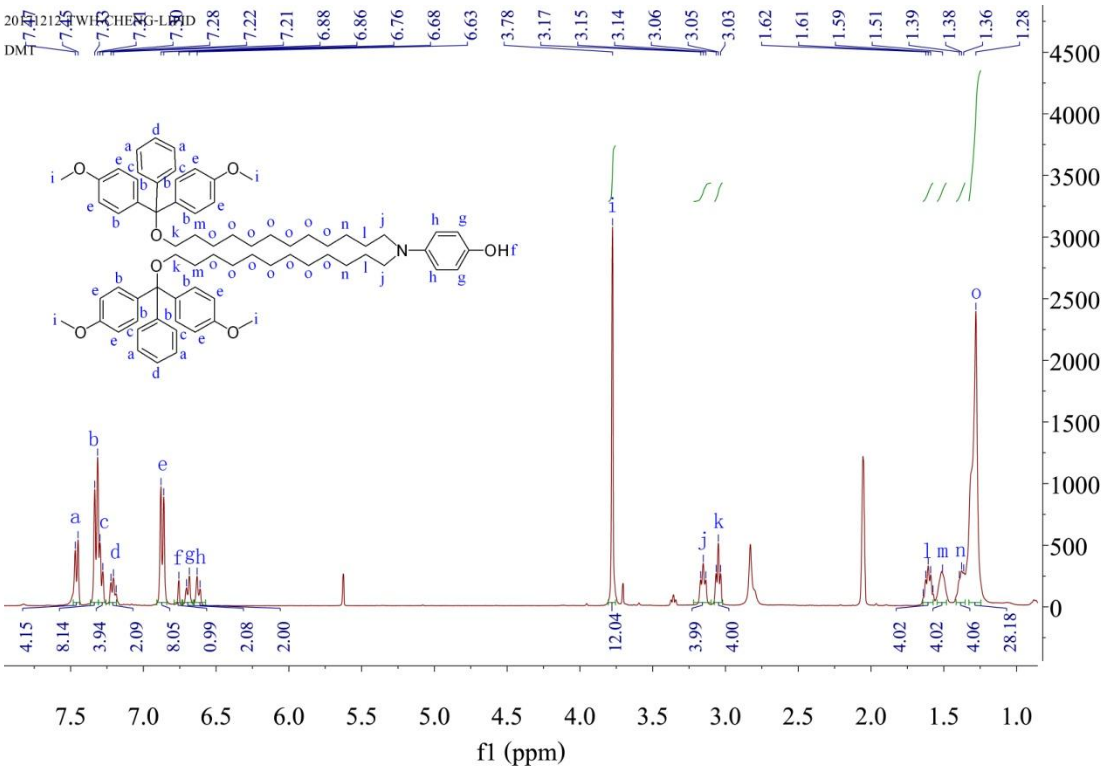

Supplementary Figure 40 ^{1}H NMR spectra of DMT-protected lipid. ^{1}H NMR peak at 5.62 (s) is the peak of residual dichloromethane; ^{1}H NMR peak at 2.05 (s) is the solvent residual peak of acetone-d6.

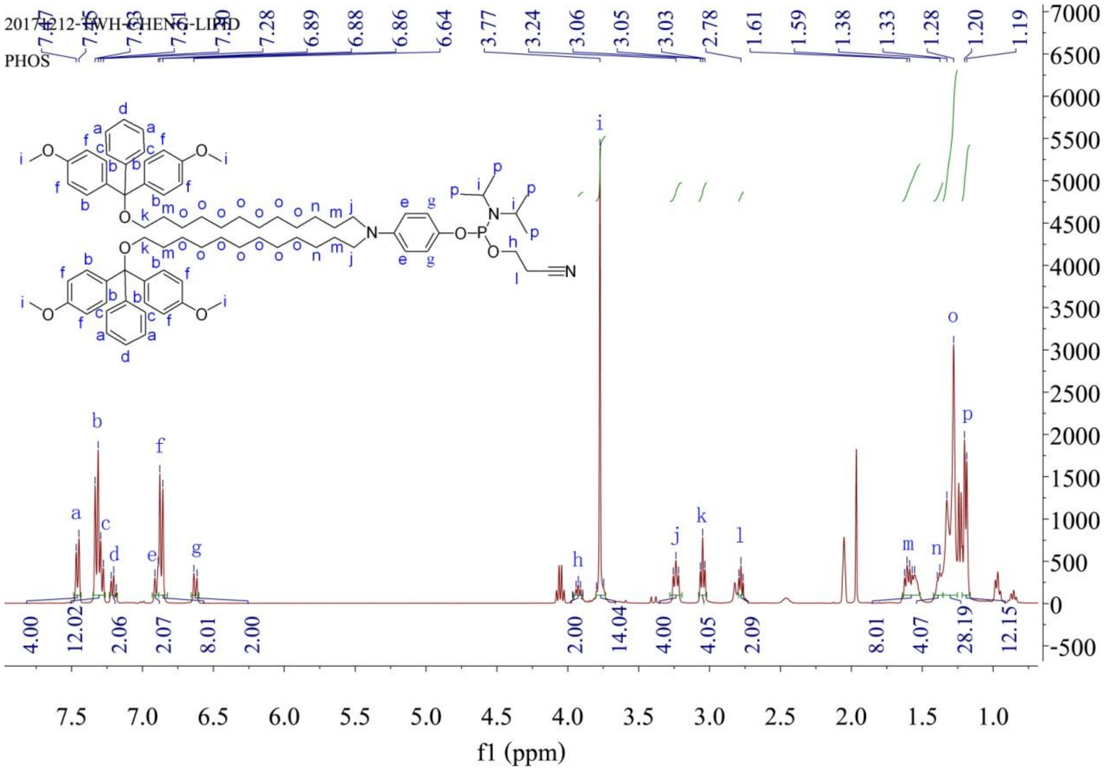

Supplementary Figure 41 ^{1}H NMR spectra of lipid phosphoramidite. ^{1}H NMR peaks at 4.05 (q, *J* = 7.1 Hz) and 1.96 (s) is the peaks of the residual ethyl acetate; ^{1}H NMR peak at 2.05 (s) is the solvent residual peak of acetone-d6.

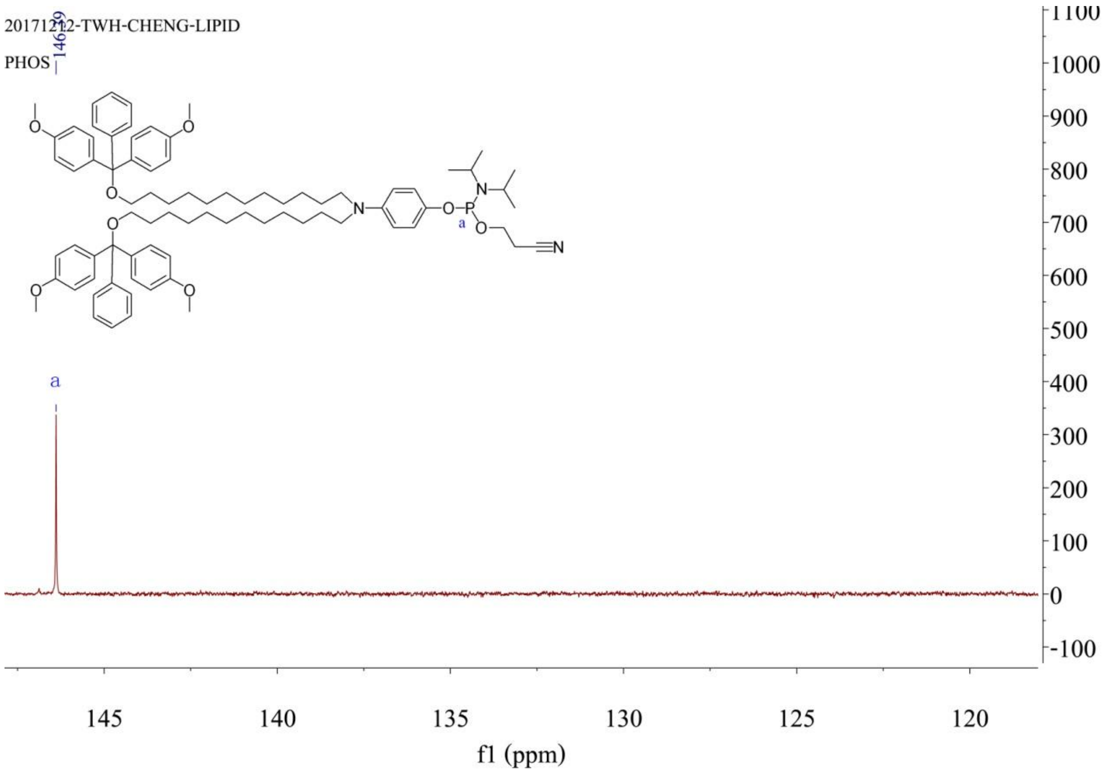

Supplementary Figure 42 ^{31}P NMR spectra of lipid phosphoramidite.

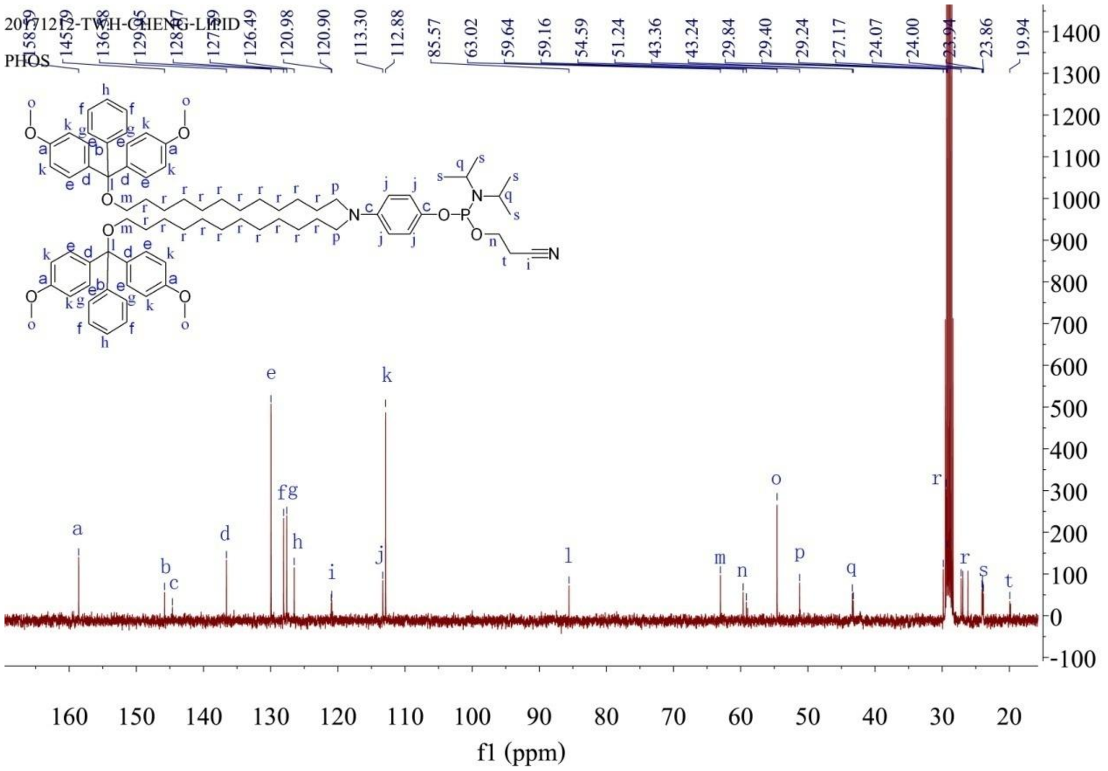

Supplementary Figure 43 ^{13}C NMR spectra of lipid phosphoramidite.

Supplementary References

1. Jin, C. et al. Floxuridine homomeric oligonucleotides "hitchhike" with albumin in situ for cancer chemotherapy. *Angew. Chem. Int. Ed.* **57**, 8994-8997 (2018).
