# Supplementary material for: Phosphorylated lipid-conjugated oligonucleotide selectively anchors on cell membranes with high alkaline phosphatase expression
Source: Nat Commun. 2019 Jun 20;10:2704. doi: 10.1038/s41467-019-10639-6 (PMC6586821; doi:10.1038/s41467-019-10639-6)
Supplement: Supplementary file 3 — Reporting Summary [file 41467_2019_10639_MOESM3_ESM.pdf]

nature research

Corresponding author(s): Weihong Tan

Last updated by author(s): Mar 9, 2019

Reporting Summary

Nature Research wishes to improve the reproducibility of the work that we publish. This form provides structure for consistency and transparency in reporting. For further information on Nature Research policies, see Authors & Referees and the Editorial Policy Checklist.

Statistics

For all statistical analyses, confirm that the following items are present in the figure legend, table legend, main text, or Methods section.

n/a	Confirmed	
☒	☐	The exact sample size (*n*) for each experimental group/condition, given as a discrete number and unit of measurement
☐	☒	A statement on whether measurements were taken from distinct samples or whether the same sample was measured repeatedly
☒	☐	The statistical test(s) used AND whether they are one- or two-sided<br>*Only common tests should be described solely by name; describe more complex techniques in the Methods section.*
☒	☐	A description of all covariates tested
☒	☐	A description of any assumptions or corrections, such as tests of normality and adjustment for multiple comparisons
☐	☒	A full description of the statistical parameters including central tendency (e.g. means) or other basic estimates (e.g. regression coefficient) AND variation (e.g. standard deviation) or associated estimates of uncertainty (e.g. confidence intervals)
☒	☐	For null hypothesis testing, the test statistic (e.g. *F*, *t*, *r*) with confidence intervals, effect sizes, degrees of freedom and *P* value noted<br>*Give P values as exact values whenever suitable.*
☒	☐	For Bayesian analysis, information on the choice of priors and Markov chain Monte Carlo settings
☒	☐	For hierarchical and complex designs, identification of the appropriate level for tests and full reporting of outcomes
☒	☐	Estimates of effect sizes (e.g. Cohen's *d*, Pearson's *r*), indicating how they were calculated

Our web collection on statistics for biologists contains articles on many of the points above.

Software and code

Policy information about availability of computer code

Data collection	For NMR data collection, the Bruker 400 MHZ Advance Spectrometer Instruction was utilized. For DNA purification and retention time analysis, Agilent 1260 Infinity HPLC was used. For quantitative analysis of oligonucleotides, Shimadzu UV-2450 Spectrophotometer was utilized. For mass spectroscopy analysis of oligonucleotides, Thermo LCQ was used (performed by Sangon Biotech (Shanghai) Co., Ltd.). For fluorescence spectroscopy measurements, FluoroMax-4 Spectrometer was utilized. For cellular binding affinity measurements, BD FACSVerse flow cytometer was used. For cell imaging, Olympus FV1000 Confocal Microscope was utilized.
Data analysis	OriginPro 8 was used to analyze the data, including retention times and fluorescence spectrum; MestReNova was used to analyze the NMR data; BD FACSuite was used to analyze the data of flow cytometry assays; FV10-ASW 2.0 Viewer was used to analyze the cell imaging data.

For manuscripts utilizing custom algorithms or software that are central to the research but not yet described in published literature, software must be made available to editors/reviewers. We strongly encourage code deposition in a community repository (e.g. GitHub). See the Nature Research guidelines for submitting code & software for further information.

Data

Policy information about availability of data

All manuscripts must include a data availability statement. This statement should provide the following information, where applicable:

- Accession codes, unique identifiers, or web links for publicly available datasets
- A list of figures that have associated raw data
- A description of any restrictions on data availability

All the Data supporting the findings of this study are available within the article and its supplementary information files.

Field-specific reporting

Please select the one below that is the best fit for your research. If you are not sure, read the appropriate sections before making your selection.

☒ Life sciences ☐ Behavioural & social sciences ☐ Ecological, evolutionary & environmental sciences

For a reference copy of the document with all sections, see nature.com/documents/nr-reporting-summary-flat.pdf

Life sciences study design

All studies must disclose on these points even when the disclosure is negative.

Sample size	No statistical methods were used to predetermine sample size. For FACS assays, samples with six different concentrations were utilized as the parallel controls.
Data exclusions	No data exclusion.
Replication	Reproducibility of the data was confirmed by at least three independent experiments.
Randomization	Samples were randomly allocated into groups.
Blinding	Not applicable, as samples were processed identically through standard and in some cases automated procedures (enzymatic dephosphorylation, FACS assays and cellular imaging analysis) that should not have bias outcomes.

Reporting for specific materials, systems and methods

We require information from authors about some types of materials, experimental systems and methods used in many studies. Here, indicate whether each material, system or method listed is relevant to your study. If you are not sure if a list item applies to your research, read the appropriate section before selecting a response.

Materials & experimental systems

n/a	Involved in the study	
☒	☐	Antibodies
☐	☒	Eukaryotic cell lines
☒	☐	Palaeontology
☒	☐	Animals and other organisms
☒	☐	Human research participants
☒	☐	Clinical data

Methods

n/a	Involved in the study	
☒	☐	ChIP-seq
☐	☒	Flow cytometry
☒	☐	MRI-based neuroimaging

Eukaryotic cell lines

Policy information about cell lines

Cell line source(s)	The human liver hepatocellular carcinoma (HepG2) and human bone osteosarcoma (U-2 OS) cell lines were purchased from Xiangya School of Medicine, Central South University, Changsha
Authentication	All of the cell lines were authenticated by short tandem repeat (STR)-profiling.
Mycoplasma contamination	No
Commonly misidentified lines (See ICLAC register)	Not applicable.

Flow Cytometry

Plots

Confirm that:

☒ The axis labels state the marker and fluorochrome used (e.g. CD4-FITC).

☒ The axis scales are clearly visible. Include numbers along axes only for bottom left plot of group (a 'group' is an analysis of identical markers).

☐ All plots are contour plots with outliers or pseudocolor plots.

☒ A numerical value for number of cells or percentage (with statistics) is provided.

Methodology

Sample preparation	HepG2 and U-2 OS cells were cultured in 6-well plates and maintained in DMEM medium supplemented with 10% fetal bovine serum and 0.5 mg/mL penicillin-streptomycin at 37 °C in 5% CO_2. Then, cells were digested from 6-well plates by using 0.2% EDTA and washed with TBS buffer twice. Then, about 300 thousand cells were incubated with the corresponding oligonucleotides at 37 °C for one hour. When incubation was completed, cells were washed twice with TBS buffer and subjected to flow cytometry. For cDNA hybridization experiments, TAMRA-labeled DNA-lipid and DNA-lipid-P were incubated with HepG2 cells for one hour. After washing twice, cells were incubated with Dabcyl-cDNA in TBS buffer for ten minutes. Then, cells were washed twice again and subjected to flow cytometry.
Instrument	BD FACSVerse flow cytometer
Software	BD FACSuite
Cell population abundance	5000 cells were collected for every assays.
Gating strategy	Not applicable.

☐ Tick this box to confirm that a figure exemplifying the gating strategy is provided in the Supplementary Information.
